# Extremely low-coverage whole genome sequencing in South Asians captures population genomics information

**DOI:** 10.1186/s12864-017-3767-6

**Published:** 2017-05-22

**Authors:** Navin Rustagi, Anbo Zhou, W. Scott Watkins, Erika Gedvilaite, Shuoguo Wang, Naveen Ramesh, Donna Muzny, Richard A. Gibbs, Lynn B. Jorde, Fuli Yu, Jinchuan Xing

**Affiliations:** 1 Department of Molecular and Human Genetics, Human Genome Sequencing Center, Baylor College of Medicine, Houston, TX 77030 USA; 2 Department of Genetics, Human Genetics Institute of New Jersey, Rutgers, The State University of New Jersey, Piscataway, NJ 08854 USA; 3 Department of Human Genetics, Eccles Institute of Human Genetics, University of Utah, Salt Lake City, UT 84112 USA

**Keywords:** Single nucleotide variant, Whole genome sequencing, South Asian, Extremely low coverage, Population structure, Imputation

## Abstract

**Background:**

The cost of Whole Genome Sequencing (WGS) has decreased tremendously in recent years due to advances in next-generation sequencing technologies. Nevertheless, the cost of carrying out large-scale cohort studies using WGS is still daunting. Past simulation studies with coverage at ~2x have shown promise for using low coverage WGS in studies focused on variant discovery, association study replications, and population genomics characterization. However, the performance of low coverage WGS in populations with a complex history and no reference panel remains to be determined.

**Results:**

South Indian populations are known to have a complex population structure and are an example of a major population group that lacks adequate reference panels. To test the performance of extremely low-coverage WGS (EXL-WGS) in populations with a complex history and to provide a reference resource for South Indian populations, we performed EXL-WGS on 185 South Indian individuals from eight populations to ~1.6x coverage. Using two variant discovery pipelines, SNPTools and GATK, we generated a consensus call set that has ~90% sensitivity for identifying common variants (minor allele frequency ≥ 10%). Imputation further improves the sensitivity of our call set. In addition, we obtained high-coverage for the whole mitochondrial genome to infer the maternal lineage evolutionary history of the Indian samples.

**Conclusions:**

Overall, we demonstrate that EXL-WGS with imputation can be a valuable study design for variant discovery with a dramatically lower cost than standard WGS, even in populations with a complex history and without available reference data. In addition, the South Indian EXL-WGS data generated in this study will provide a valuable resource for future Indian genomic studies.

**Electronic supplementary material:**

The online version of this article (doi:10.1186/s12864-017-3767-6) contains supplementary material, which is available to authorized users.

## Background

The rapid development of next-generation sequencing technologies has resulted in a fruitful decade of genomic discoveries, many of which are becoming integrated into translational settings and promise to dramatically improve clinical outcomes [1]. Despite the tremendous reduction in sequencing costs and increase in data generation throughput, projects that require interrogating more than a few hundred human genomes can still be costly. Efforts that explored a “low coverage” sequencing strategy, such as the 1000 Genomes Project [2] and the CHARGE Project [3], sequenced thousands of human subjects at 4–10x read depth of coverage each. These projects have been very successful by leveraging informatics algorithms and imputation methods to achieve variant discoveries with exceedingly high quality and sensitivity [4]. In addition, several studies have demonstrated that genotype likelihood and read information from low-coverage sequencing can be directly used for population genetics analyses, without genotype calling [5–7].

We aim to continue these developments and hypothesize that a study design of sequencing at population scale (e.g., more than a few hundred subjects) with each subject at 1–2x coverage (i.e., extremely low coverage) would capture sufficient information to understand population genomic attributes such as diversity, population substructure, and admixture. Such a study design would decrease the cost for a population genomics study to tens of thousands of dollars. This design has broad utility for both population genomics studies in various model species [8] and disease genetics studies [9, 10]. For example, while projects such as the 1000 Genomes Project and the HapMap Project [11, 12] surveyed a number of populations to generate a large resource of reference panels, populations or ethnicities that have large genetic distances from these sample panels have poor imputation power using these standard reference panels [13]. An extremely low coverage, population-level WGS surveys would provide additional information for these populations.

South Asian populations are an example of major populations that lack adequate reference panels. South Asian populations on the Indian subcontinent are known to have a complex demographic history with multiple socio-linguistic groups [14, 15]. There is also evidence of founder effect [16] and a long history of endogamy along caste and tribal lines [16, 17], making South Asian populations among the most diverse populations with unique disease profiles [18, 19]. Association studies among South Asians, using generic genotyping arrays and reference panels for interrogation and imputation, could fail to identify associated loci specific to these populations due to their unique genetic variants and haplotypes. Many South Asian populations sequenced in previous studies [2, 20], including the 1000 Genomes Project, were migrant populations and captured only a limited amount of the genetic variation and haplotype diversity present in the Indian subcontinent. Our proposed strategy holds potential for surveying various South Asian populations to catalogue new genetic variation at an affordable cost.

As a proof-of-principle, we present the results from extremely low coverage whole genome sequencing (EXL-WGS) of eight South Asian populations from a wide spectrum of social and cultural strata living in the state of Andhra Pradesh. The population samples belong to four broad self-identified classifications: lower caste, middle caste, upper caste and tribe. Using EXL-WGS of 185 samples with coverages between 1x and 2x, we demonstrate that the EXL-WGS study design generates accurate genomic variant information and reliably recapitulates population substructure generated by previous methods. Moreover, sequencing approaches provide an advantage for population studies compared to genotyping array platforms, which are known to have substantial ascertainment bias [21, 22]. Furthermore, we show that the genotypes from EXL-WGS can serve as a customized reference panel, adding more power to association studies than existing genomic data [2].

## Results

### Samples and sequencing coverage

We sequenced 185 samples from the state of Andhra Pradesh to an average read depth of coverage of 1.6x (ranging from 0.84x to 3.39x) (Additional file 1: Table S1). We refer to our dataset as SAS-AP (South Asian-Andhra Pradesh). The cohort consists of eight populations across four social strata (Table 1). On average, ~65% (standard deviation (stdev) 7%) and ~38% (stdev 9%) of the genome is covered by at least one read or two reads, respectively. At 1.6x coverage, 77 and 47% of the genome is predicted to be covered by at least one read and two reads, respectively, based on the Lander-Waterman statistics [23] (Additional file 2: Figure S1.1). Our result is comparable to the expectation.

**Table 1:** Sequencing and variant calling statistics for SAS-AP samples

Group	Populations	# of samples	Depth of Coverage	Total SNVs	Avg SNVs	Ti/Tv	Novel 1000G-SAS	Novel dbSNP 141
^a^Upper	Brahmins	16	1.81	4,443,583	2,571,972	2.09	97,022	5632
^a^Middle	Kapu	37	1.57	4,457,414	2,582,833	2.09	97,303	5701
^a^Middle	Yadava	32	1.65	4,457,237	2,575,646	2.09	97,300	5699
^a^Lower	Mala	23	1.56	4,455,626	2,615,726	2.09	97,261	5701
^a^Lower	Madiga	24	1.35	4,455,972	2,583,914	2.09	97,271	5699
^a^Lower	Relli	15	1.69	4,455,972	2,586,868	2.09	96,908	5639
Tribal	Irula	22	1.86	4,439,932	2,570,388	2.09	97,024	5674
Tribal	Khonda Dora	16	1.82	4,403,761	2,574,472	2.09	96,340	5523
Total		185	1.64	4,457,475	2,583,004	2.09	97,309	5701

Only SNVs with minor allele frequency (MAF) ≥10% are included. Total SNVs: the total number of SNVs in a population. Avg SNVs: the average number of SNVs in an individual. Ti/Tv: transition/transversion SNV ratio. Novel 1000G-SAS: the number of SNVs that are not in the 1000G-SAS dataset. Novel dbSNP 141: the number of SNVs that are not in dbSNP 141. ^a^Caste Populations

### Variants discovery

To test the feasibility of an EXL-WGS sequencing design for variant identification, we first simulated a cohort of 208 samples from the African populations in the 1000 Genomes Phase 1 data (see Protocol 1 for details (Additional file 2: Section S2)). The coverages in the simulated dataset closely matched that of the SAS-AP dataset (Additional file 2: Section S2). When the variance ratio statistic parameter (*s*) is set at 2.8, SNPTools [24] recovered all single nucleotide variants (SNVs) with minor allele frequency (MAF) ≥ 10%. The false discovery rate was bounded by 3%, where the majority of false positive sites had a MAF < 10%. The average individual genotype discordance rate for SNVs with MAF ≥ 10% was 6.43% (stdev 4.93%). This result demonstrates that SNPTools has good accuracy in calling SNVs with MAF ≥ 10% using EXL-WGS data. Therefore, we identified SNVs in the SAS-AP dataset using SNPTools with *s* = 2.8 as determined in the simulation study (Additional file 2: Section S2).

To further improve the variant calling quality, we used a second variant calling tool, the Genome Analysis Tool Kit (GATK) [25], for variant identification. Using the same simulation strategy, we generated a new set of simulated samples from the same 208 African samples on chromosome 20 and evaluated the performance of SNPTools and GATK, as well as the consensus of the two call sets. For sites with MAF ≥ 10% in the 1000 Genomes dataset, SNPTools call set has a sensitivity of 98.8% and a FDR of 19.6%. GATK called 116,348 variants with MAF ≥ 10%, with a sensitivity of 88.7% and a FDR of 16.5%. The consensus of the two pipelines yielded a recall rate of 88.5%, while reducing the FDR to ~13% (Additional file 2: Figure S2.4). Because the consensus call set improves the FDR with only a small reduction in sensitivity, we called variants in SAS-AP data using the same approach and autosomal biallelic SNVs with MAF ≥ 10% were selected as our final call set and used for all subsequent population genetics analyses.

The final call set included 4,457,475 autosomal biallelic SNVs with a transition/transversion ratio of 2.09. Of these, 97,309 (2.18%) are novel with respect to the South Asian dataset in the 1000 Genomes Project Phase 3 (1000GP3-SAS), and ~5700 SNVs are absent in dbSNP build 141. The vast majority of the SNV sites have an average coverage between 0.5x and 4x, close to our average sequencing depth of 1.6x (Additional file 2: Figure S4.1). The number of SNVs in each sample ranges from 2,485,817 to 2,930,235, with an average of 2,583,005 (stdev 37,370) (Additional file 1: Table S1).

To assess the quality of the EXL-WGS call set, we compared the EXL-WGS calls to single nucleotide polymorphism (SNP) genotyping array genotype calls and Sanger sequencing results from previous studies [18, 26]. For 42 of the samples that were previously genotyped using SNP arrays [26], 93.2% of the SNVs were recovered by EXL-WGS dataset. In another study, 63 samples in this study were sequenced for a 100 Kb ENCODE region on chromosome 12 with Sanger sequencing [18] (referred as ENCODE data set hereafter). Within the 100 Kb region, 75.3% of the SNVs were rediscovered by EXL-WGS dataset (Additional file 2: Section S3). Among the 4301 heterozygous calls in the ENCODE dataset, 4231 (98.4%) were correctly called by SAS-AP dataset (Additional file 2: Section S4.2). In particular, we achieve high genotyping accuracy (97.3%) even for heterozygous sites that have no coverage in an individual.

### Population genetic analyses

Next we assessed if the EXL-WGS provides sufficient information for examining population structure in SAS-AP samples. First, simulation experiments were carried out by thinning the reads from the 1000 Genomes dataset to test the feasibility of performing principal component analysis (PCA) on common SNVs in an EXL-WGS cohort (Additional file 2: Section S2.2). PCA was carried out on four simulated datasets with coverages of 0.25x, 0.5x, 0.75x and 1x, respectively (Additional file 2: Figure S2.3). Simulation results suggest that SNVs with MAF > 20% are sufficient to detect population structure by PCA in all four call sets.

**Fig. 1:**
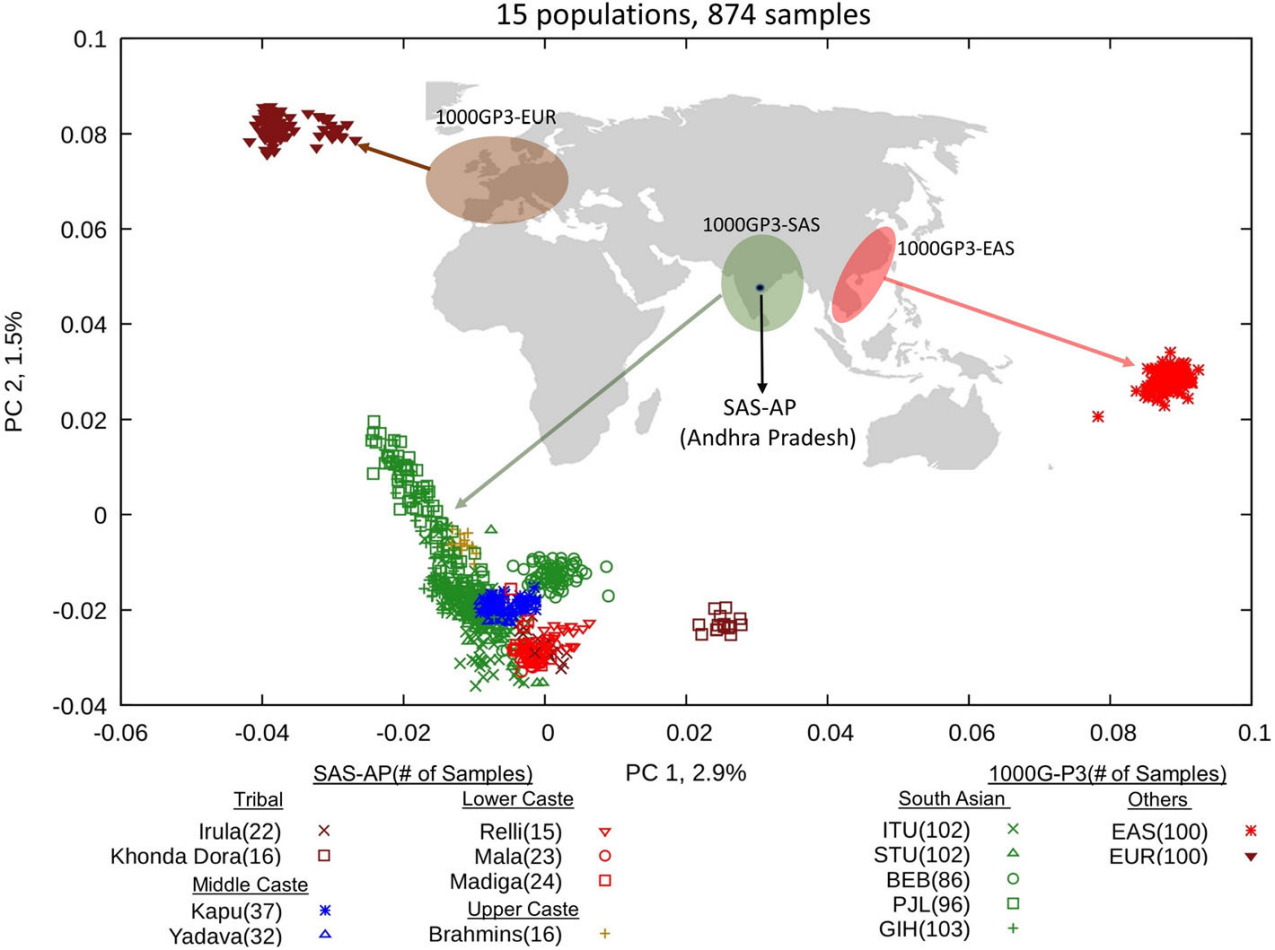
PCA of SAS-AP and 1000GP3 samples. Each symbol represents one individual. PC1 and PC2 are shown on the X and Y axis, respectively. The percentage of variance explained by each PC is labeled on the axis. The map shown in the figure is adapted from https://commons.wikimedia.org/wiki/File:World_map_blank_without_borders.svg where permission is granted under a creative commons license

Using SAS-AP variant calls with MAF ≥ 10%, we examined the relationship among the populations/groups in our dataset. PCA of SAS-AP samples with the 1000 Genomes Project Phase 3 European (1000GP3-EUR), East Asian (1000GP3-EAS) and South Asian (1000GP3-SAS) samples showed that our samples are most closely related to the 1000GP3-SAS samples, as expected (Fig. 1). The Khonda Dora samples cluster separately from all other south Asian samples on principal component 1 (PC1, variance = 2.9%) and are the closest to 1000GP3-EAS samples among south Asian samples (Fig. 1). When the 1000 Genomes Project Phase 3 African and American samples are included, the SAS-AP samples still clustered with 1000GP3-SAS samples (Additional file 2: Figure S5.1). Consistent with the PCA result, F_ST_ statistics place SAS-AP closest to 1000GP3-SAS. Except for Khonda Dora, all other populations are closer to 1000GP3-EUR than 1000GP3-EAS (Additional file 2: Table S5.1). The 1000GP3-AFR populations have the largest distance with all SAS-AP populations, which is consistent with the out-of-Africa expansion model.

**Fig. 2:**
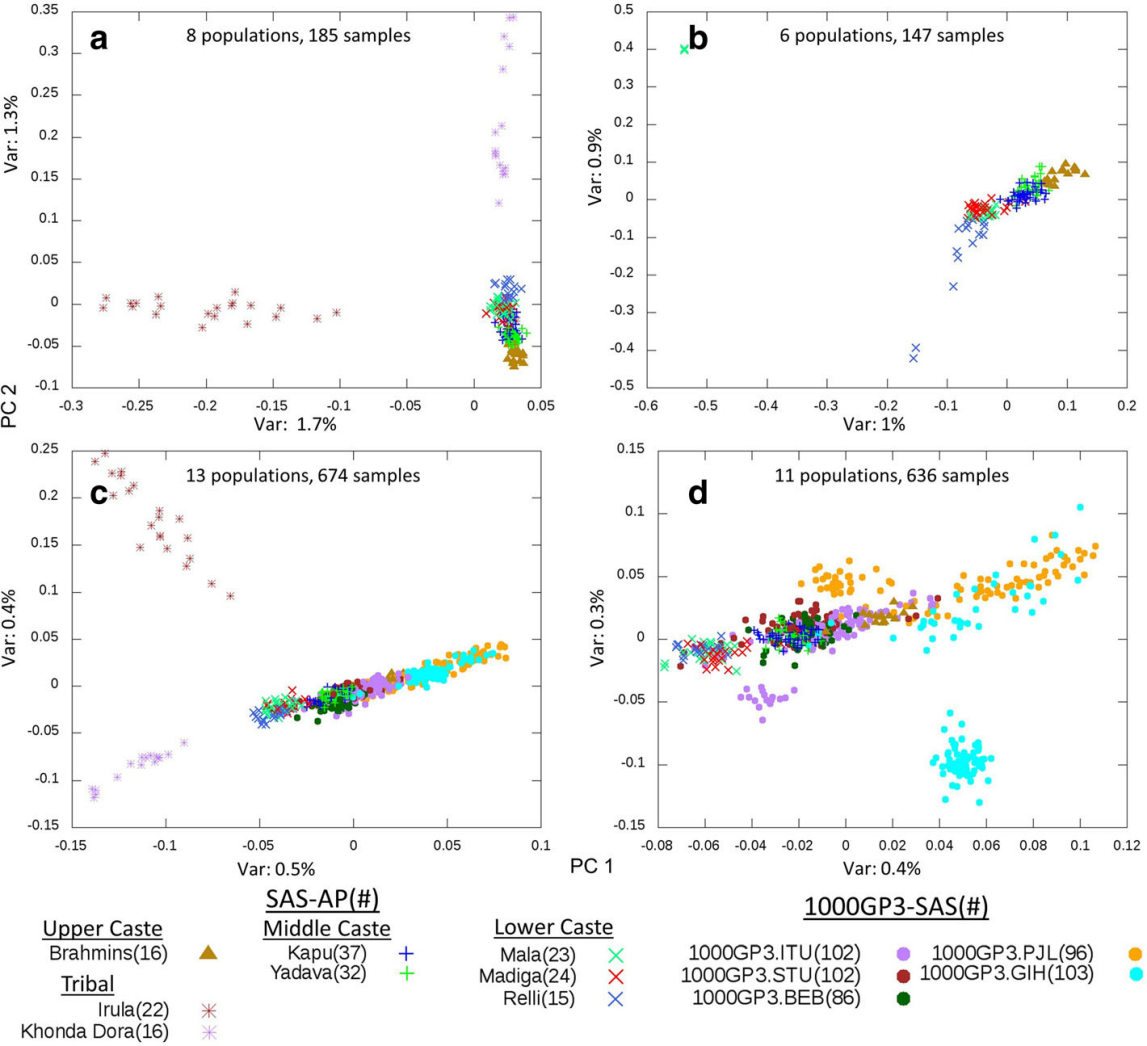
PCA of South Asian samples. **a** All SAS-AP samples; **b** SAS-AP excluding Khonda Dora and Irula samples; **c** SAS-AP and 1000GP3-SAS samples; **d** SAS-AP and 1000GP3-SAS excluding Khonda Dora and Irula samples. Each symbol represents one individual. PC1 and PC2 are shown on the X and Y axis, respectively. The variance explained by each PC is labeled on the axis

To further examine fine-scale population structure within our samples, we performed PCA on SAS-AP samples only (Fig. 2a). The two tribal groups, Irula and Khonda Dora, are clearly separated from the caste groups on PC1 and PC2, respectively. Consistent with this result, Irula has the highest pairwise F_ST_ with all other SAS-AP group, followed closely by Khonda Dora, except for Brahmins, who have a higher F_ST_ with Khonda Dora than Irula (Additional file 2: Table S5.2). The mean pairwise F_ST_ of populations in SAS-AP is 0.015 (stdev 0.012). This value is higher than those of other populations sampled across the Indian subcontinent (0.0109, [16]). PCA that includes the 1000GP3-SAS samples also shows distinctive clustering of Khonda Dora and Irula tribal samples separated from the caste samples (Fig. 2c). While the non-tribal populations are not as clearly differentiated as the tribal groups, there is evidence of clustering along caste-based lines. When PCA is performed on caste samples only (Fig. 2b, d), lower caste samples cluster separately from 1000GP3-GIH (Gujaratis in Houston), 1000GP3-PJL (Punjabis from Lahore), and Brahmins on PC1. Lower caste samples are also mostly separate from 1000GP3-BEB (Bengalis from Bangladesh), 1000GP3-ITU (Telugu from UK) and 1000GP3-STU (Sri Lankan Tamils from UK) samples (Fig. 2d). Middle caste Yadava and Kapu samples are indistinguishable from the 1000GP3-BEB, ITU and STU samples but can be distinguished based on PC1 values from the lower caste, upper caste, 1000GP3-GIH, and 1000GP3-PJL samples. The upper caste Brahmin samples are differentiated from lower caste and tribal samples in all PCA plots and are the closest to 1000GP3-PJL and 1000GP3-GIH samples. This is consistent with previous research suggesting a larger west Eurasian genetic component in upper castes compared to other castes [16, 27].

**Fig. 3:**
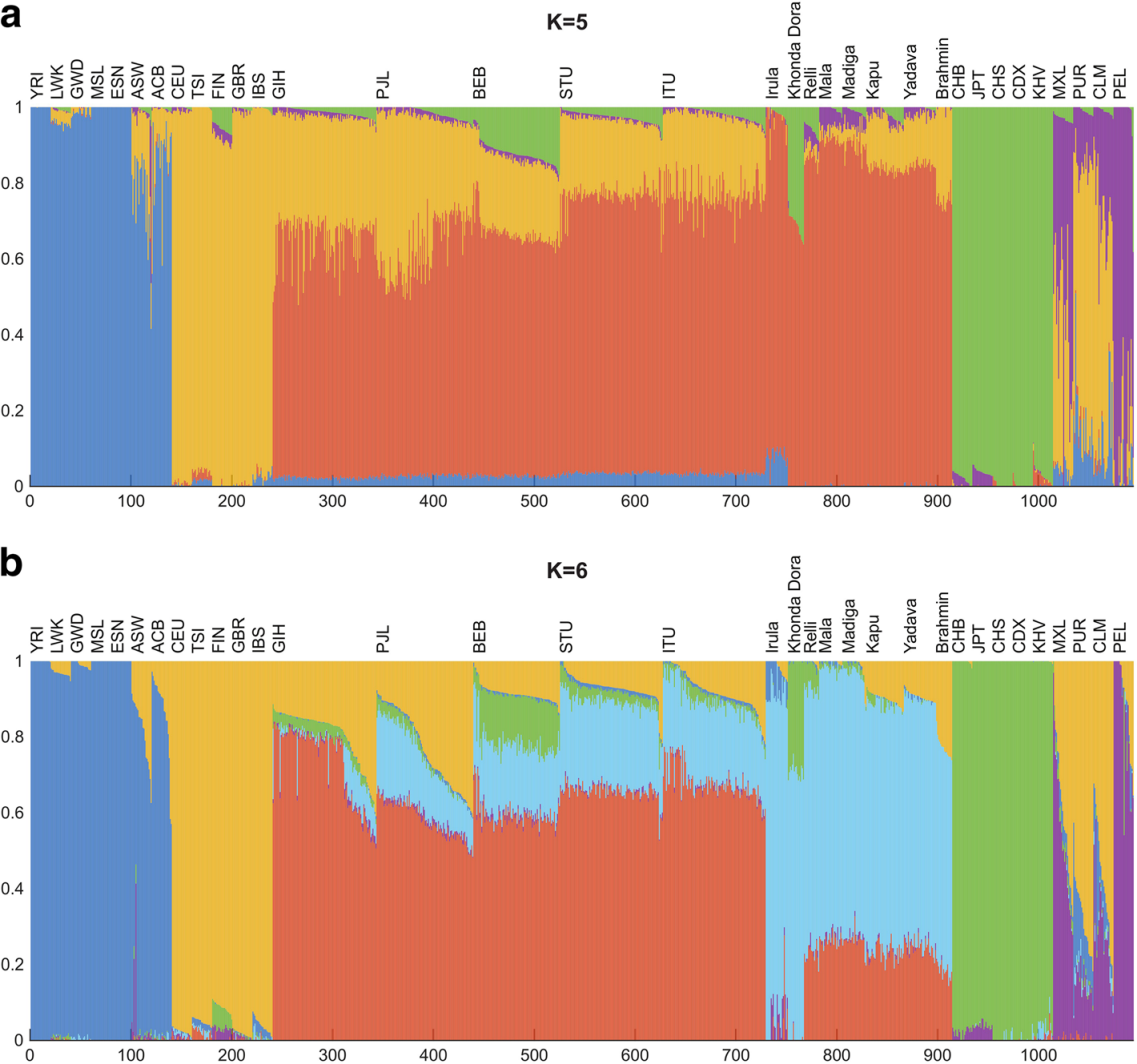
Admixture analysis of SAS-AP and 1000GP3 samples. **a** K = 5; **b** K = 6. Each vertical bar represents one sample. The vertical bar is composed of colored sections, where each section represents the proportion of a sample’s ancestry derived from one of K ancestral populations

Next, we examined the composition of potential ancestral components in SAS-AP and 1000GP3 samples using the *ADMIXTURE* program [28] (Fig. 3, Additional file 2: Figure S5.2). At K = 4, four ancestral components corresponding to Africa, Europe, India, and East Asia were identified (Additional file 2: Figure S5.2). At K = 5, the five ancestral components corresponded to the major continental groups: Africa, Europe, India, East Asia, and America (Fig. 3a). At K = 6, two groups within India were identified: one is predominantly represented in the 1000GP3 samples, and one in the SAS-AP samples (Fig. 3b). Previously studies have also identified two similar main ancestral groups in India and termed them “Ancestral North Indians” (ANI) and “Ancestral South Indians” (ASI) [16]. Most of our SAS-AP samples contain an admixture of ANI and ASI components, with the majority of the predicted ancestry from ASI. Interestingly, compared to the caste groups, the two tribal groups showed distinct ancestry: Irula samples are dominated by the ASI component while Khonda Dora samples have a distinctively large (>20%) East Asian ancestral component compared to other SAS-AP samples. It is also notable that at K = 6, the 1000GP3 Finnish population has more Asian and American-like components than do other Europeans. This might be explained by Finnish origins: many Finns are thought to have ancestry from southeastern Europe and share ancestral components with Asian/American people [29, 30]. At K = 7, an ancestral group that is dominant in Irula samples is recognized (Additional file 2: Figure S5.2).

Several recent studies have proposed to directly use genotype likelihood (GL) from low-coverage sequencing for population genetics analyses, without genotype calling [5–7]. For sites covered by sequencing reads, using GL before calling genotype should maintain more information for population genetics analysis. We compared the population genetic analysis results for genotype-based analyses with GL-based analyses (Additional file 2: Section S5.2). The PCA, Admixture, and F_ST_ results for the two types of analyses showed similar results in general. The GL-based PCA showed a tighter clustering of the samples than the genotype-based PCA but the overall pattern and the amount of variance explained are similar between the two plots (Additional file 2: Figure S5.3). This observation is consistent with the original study where genotype-based PCA using common variants are similar to GL-based PCA [5].

### Imputation performance

The EXL-WGS study design can be a highly effective and affordable strategy to generate population-specific imputation reference panels, which can improve imputation accuracy in association studies that use SNP arrays as primary data sources. Using a simulation dataset, we showed EXL-WGS imputation reference panel has a comparable performance to the SNP array reference panel within the same population (Additional file 2: Section S6). However, when the population of interest has a large genetic distance from the available reference panels, EXL-WGS could provide a better imputation panel than a generic reference panel. To test this hypothesis, we examined whether imputation accuracy can be improved by creating a population-specific reference panel using SAS-AP samples than using the 1000 Genomes South Asian reference panel. The weighted F_ST_ estimates between populations in SAS-AP and 1000GP3-SAS is maximum for tribal populations at approximately 0.02.

**Fig. 4:**
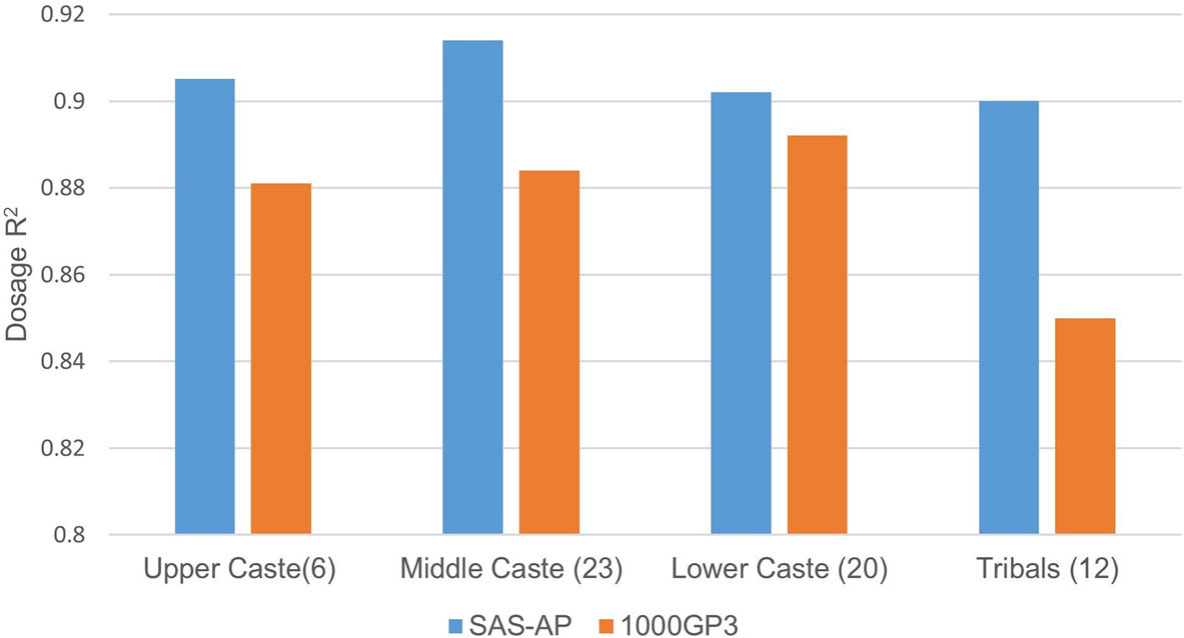
Imputation dosage correlation coefficient R^2^ of ~200,000 missing sites from SAS-AP samples. Results from the SAS-AP reference panel and the 1000GP3-SAS reference panel are shown in *blue* and *red bars*, respectively. The number of target samples is given in parenthesis

For the imputation experiment, approximately one-third of the samples from each of the main caste and tribal classifications from SAS-AP were chosen as a target set for imputation. The remaining samples from SAS-AP were used as a representative EXL-WGS population-specific reference panel, and 160 randomly selected 1000GP3-SAS samples were used as the generic reference panel. Approximately 5% of sites were removed from the target set (see Methods) and the performances of the two reference panels are compared. For SNVs removed in the target site, the custom SAS-AP reference panel had a higher dosage correlation coefficient (R^2^) value than the 1000GP3 panel for all population classifications (Fig. 4). The R^2^ is most pronounced (0.90 vs 0.85) in the tribal population and least apparent for the lower caste (0.902 vas 0.892). All the missing SNVs were recovered using both the reference panels. Given the high genetic diversity in the Indian subcontinent, and the unique ancestry profiles of populations, using a custom reference panel will be better than using any of the existing populations, even for SNVs in the MAF range ≥ 10%.

To determine if the better performance of the SAS-AP reference panel is due to the batch effect between the 1000 Genomes and the SAS-AP samples, or due to population composition, we further evaluated the performance of the reference panels for imputing the 1000 Genomes ITU samples as the target (Additional file 2: Section S7). Imputation results from different 1000 Genomes Indian samples suggest the reference panel population composition has a considerable effect on the reference panel performance. For example, the 1000 Genomes reference panel from ITU samples outperformed SAS-AP samples for ITU sample imputation, as expected (Additional file 2: Figure S7.1). However, SAS-AP panel performed as well as the reference panel from the 1000 Genomes GIH, PJL, and BEB samples (Additional file 2: Figure S7.2). This result highlights the need of an extensive sampling of Indian populations.

### Mitochondrial genome

Because of the high copy number of mitochondrial DNA, EXL-WGS should yield higher coverage for the mitochondrial genome than for the single-copy nuclear genome. To test this hypothesis, we determined the depth of coverage for the 185 mitochondrial genomes (Additional file 1: Table S1). All nucleotide bases in all samples were covered by at least one read, and on average, 99.94% percent of bases had >10x coverage. Mean depth of coverage ranged from 57x to 266x, with an average coverage of 124x for all samples.

Using the EXL-WGS data, we generated a high-quality mitochondrial genome sequence for each sample. To further assess the EXL-WGS quality, we generated mtDNA sequence data for sample I8 and KD7 on an Ion Torrent PGM and compared the results to the Illumina EXL-WGS data. For both samples, all base calls between the PGM and EXL-WGS were in agreement except for one difference in I8 at position 3107 (N vs. T) and four single C base addition in KD7. All four additions in KD7 are within homopolymeric C regions. These differences are likely attributable to the well-known difficulties in sequencing homopolymeric regions on the Ion Torrent platform. Additionally, no differences were found between the EXL-WGS data and lineage-defining SNVs genotyped previously in a subset of the 185 samples using single-base extension genotyping [31]. Taken together, these results demonstrate that EXL-WGS produces high-quality complete mitochondrial genome sequence data.

**Fig. 5:**
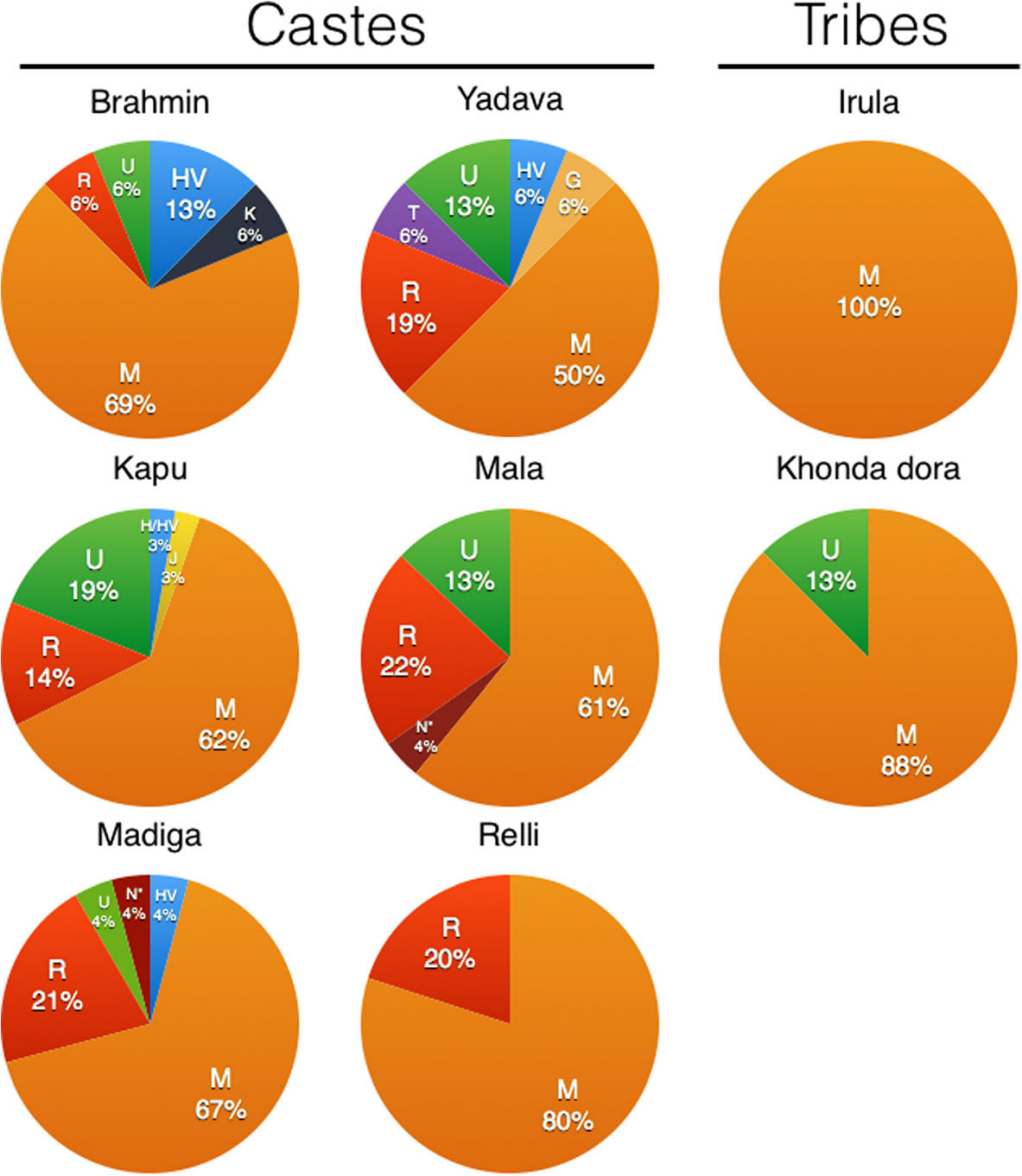
The proportion of major mtDNA haplogroups in castes and tribal populations. All haplogroups are defined using complete mtDNA sequences

Next, we determined the mtDNA haplogroup distribution among SAS-AP samples using mitochondrial whole-genome sequences. Samples were grouped into populations based on their caste or tribe affiliation, and the proportion of each major mtDNA lineage was calculated for each population (Fig. 5). Mitochondrial haplogroup M is the predominant lineage in all populations. A greater proportion of R, U, and H/HV occur in caste than in tribal populations. Caste populations, with the exception of Relli, have at least 30% non-M lineages. In contrast, non-M lineages were not observed in Irula, an isolated tribal group from southern Andhra Pradesh. Similarly, only one major non-M lineage (U) was seen in Khonda Dora, an isolated tribe from northeastern Andhra Pradesh. The population distributions of the major mtDNA haplogroups are consistent with higher gene flow and admixture into caste populations than into tribal groups. These results are concordant with previous analyses of these caste and tribal samples using Sanger-sequenced mtDNA HVS1 and lineage-defining SNVs [27, 31] and demonstrate the feasibility of generating high-quality mitochondrial genomes using EXL-WGS.

## Discussion

In the past, SNP genotyping arrays have been used to survey genomic diversity in previously unexplored populations [12, 26, 32]. With the ever-decreasing sequencing cost, EXL-WGS (e.g., ~1x) provides an alternative, and for some study designs, better approach for studying population diversity than SNP arrays. The primary benefits of EXL-WGS over SNP arrays include discovering population-specific variants for analyzing fine-scale population structure, improving imputation power, and providing high-quality mitochondrial genomes. In addition, even at current sequencing costs, the cost of EXL-WGS is comparable to or even lower than SNP arrays.

### Fine-scale population structure

SNP genotyping arrays are usually designed using existing genetic information. Therefore, for populations that have not been studied extensively and do not have a good reference population, the SNP array is likely to miss population-specific variants and bias population structure analysis [21, 22]. An accurate population structure/ancestry analysis is not only important for population genetic and evolutionary genetic studies, it is also crucial for association studies [33, 34].

Here we demonstrate the benefit of EXL-WGS over SNP genotyping arrays by studying populations from South Asia. The Indian subcontinent has witnessed multiple waves of migration since the first migration of modern humans out of Africa [35–37], which resulted in a highly variable genetic diversity among South Asian populations [18]. We were able to examine the fine-scale population structure using 4,457,466 SNVs with MAF ≥ 10% among SAS-AP samples. There were ~97,000 common variants that are not present in the 1000GP3-SAS dataset and ~5700 variants that are absent in the dbSNP database. Our PCA result suggests that SAS-AP tribal populations are distinct from caste populations (Fig. 2a, c), and the caste samples are roughly clustered along the caste groups but the clusters are less distinguishable than tribal groups (Fig. 2b, d). The *ADMIXTURE* analysis suggests South Asian samples can be predominantly divided into two clines, one aligned with populations having west Eurasian ancestry and one aligned with populations having East Asian ancestry. In our analysis, all castes are closer to the Eurasians than to the East Asians along these clines. The position of caste populations along the west Eurasian cline is consistent with previous results on higher ANI component in higher ranks [27]. The Khonda Dora population is clearly more aligned with the East Asian cline, supporting a recent report about an East Asian ancestry component in Indian tribal populations [17]. The Irula population has a predominantly ASI profile making them distinct from both the west Eurasian and East Asian clines (Fig. 3). Our results present an unbiased estimate of genetic diversity in South Asian populations and demonstrate the EXL-WGS design can be used to study the population structure.

In addition to the genotype based population genetic analyses, we also tested the methods that are designed for low-coverage WGS and directly use genotype likelihood for analysis. The results of PCA, admixture, and F_ST_ analyses are largely congruent between the two sets of analyses (Additional file 2: Section S5.2). Compared with the GL-based methods, calling genotypes with our current pipeline has different advantages and utilities. For example, for multi-individual variant calling, SNPTools can leverage linkage association information and impute genotypes for sites that have no variant reads or sequence coverage. So the number of genotype calls from SNPTools is higher than the number of sites with sequence coverage. In our comparison with the gold standard ENCODE dataset the genotype calls at sites with no sequencing coverage has a high accuracy (>97%) in our dataset (Additional file 2: Figure S4.2). In addition, for studies that need to combine data from different types of technologies, it is more straight-forward to combine genotypes.

Several other programs, such as Beagle [38] and STITCH [39], can also perform imputation on low-coverage samples without a reference panel, similar to SNPTools we used in this study. However, Beagle is optimized for use in a setting with available reference panels, and we have also demonstrated the efficacy of Beagle in this respect in this study. STITCH is optimized for read based haplotype construction for imputation and phasing. In comparison, the SNPTools imputation engine is independent of read length and is optimized for genotyping variants. This approach would allow a researcher to maximize the value of EXL-WGS data even with short read lengths.

### Enhanced imputation reference panel

Another advantage of EXL-WGS over SNP arrays is the improved power in imputation. Many populations in the Indian subcontinent have been found to have founder events, resulting in a higher burden of recessive diseases [16]. In this situation, imputation strategies using existing reference panels can be ineffective for gene mapping studies involving unique recessive variants [16, 40]. However, with an appropriate reference panel, even coverages as low as 0.01x may be sufficient to achieve more than 90% of the effective population size surveyed by a dense one million site SNP array designed for variants with MAF ≥ 5% [10]. As shown in our result, the EXL-WGS reference panel improves the imputation power over a generic 1000GP3-SAS reference panel, even though the 1000GP3-SAS samples were also from the Indian subcontinent. Moreover, our methodology of producing reference panels, can be used for using off target reads from large existing cohorts of whole exome sequencing datasets, many of which have read lengths less than 100 bp [41, 42].

### High quality mitochondrial genome

Lastly, EXL-WGS allows the interrogation of mitochondrial genomes of the sequenced samples. Even with ~1.5x coverage EXL-WGS design we obtained high-coverage mitochondrial genome sequence (average coverage 124x) and generated high-quality mitochondrial haplotypes for every SAS-AP sample. Using the haplotypes we were able to examine the material lineage diversity among the samples. Among tribal samples, all Irula people have the deep-rooted ancestral mitochondrial haplogroup M that is typical of South Asia. Thirteen percent of the Khonda Dora samples have the mitochondrial haplogroup U. Although haplogroup U is shared with western European populations, it has some deep (>35,000 years before present) India-specific branches [43]. The mitochondrial haplogroup K is only observed in the Brahmin populations, and it is estimated that this haplogroup arose in west Eurasia within the last 12,000 years [44]. These results are consistent with previous studies and demonstrate EXL-WGS can be used for high-resolution mitochondrial haplotype studies.

### Feasibility and necessity of WGS

In our project the average yield of SNVs per individual is approximately 2.5 million for MAF ≥ 10%, and a SNP genotyping array of comparable yield will cost ~ $170/sample. The cost of sequencing one gigabase has been estimated to be less than $30 on a HiSeq 2500 [45]. Accounting for library preparation costs of about $30 per sample [10], the total cost of sequencing a genome with 1.5x coverage is less than $180. Advances in library preparation methods can lead to further cost reductions of almost half [46].

For coverages less than 2x, there is a valid concern about genotyping accuracy, especially for heterozygotes. In a recent case-control study involving more than 10,000 samples with an average depth of coverage ~1.7 x, mean genotype concordance was better than 98% with respect to deep WGS gold standard datasets and SNP arrays [9]. Even though more than 5000 cases were used by [9] to replicate signals for common variation, coverage as low as 0.1x may be sufficient to infer fine-scaled ancestry information among worldwide continental populations [47]. Indeed, with multiple sample joint-calling and genotyping using SNPTools, followed by SNPTools imputation, we achieved excellent calling accuracy for heterozygous SNVs (Additional file 2: Section S4.2). Even for sites that have no coverage in an individual, we achieved high genotyping accuracy (97.3%) for heterozygous SNVs. Our results are consistent with previous studies and demonstrate the power of EXL-WGS design in leveraging genetic information at the population level.

## Conclusions

The advances in sequencing technologies are making EXL-WGS a more cost-effective and advantageous strategy than SNP genotyping arrays for studying new populations. EXL-WGS allows for the discovery of population-specific variants that are not present on a SNP array, provides a population-specific reference panel for imputation, and generate a high-quality mitochondrial genome for each sample.

## Methods

### Sample collection and sequencing

A total of 235 samples were collected from the state of Andhra Pradesh in India. All samples belong to the Dravidian language family and were collected as unrelated individuals as described previously [48, 49]. All studies of South Indian populations were approved by the Institutional Review Boards of the University of Utah and Andhra University, India.

Whole genome sequencing using DNA samples from the blood was performed at the Human Genome Sequencing Center, Baylor College of Medicine using Illumina HiSeq following a standard protocol [50]. The raw sequencing data were subject to QC and aligned to the human reference genome (hg19) using BWA [51] through the Mercury Pipeline [52]. The alignment files were then used for variant calling using SNPTools [24] and GATK [25] pipeline.

### Variant calling – SNPTools

The parameter fitting of the SNPtools pipeline was done on a simulated cohort generated using Protocol 1 from 208 AFR samples from the 1000 Genomes Phase 1 data [53] with coverages corresponding to the real data (See Additional file 2: Section S2.1 for more details). The call set was phased and imputed using SNPtools.

### Variant calling – GATK

Variant discovery by the Genome Analysis Tool Kit (GATK, v2.4-9) pipeline roughly followed the best practice recommendation for alignment processing and variant calling [54]. Starting from sorted and indexed individual BAM files, a series of GATK alignment-processing procedures were conducted, including indel realignment, PCR duplicate removal, and base quality score recalibration. Then, a joint genotype calling was performed on all individuals with GATK UnifiedGenotyper to generate the raw genotype call in a single variant-calling format (VCF) file. The quality scores were then recalibrated with VariantRecalibrator according to the GATK recommended parameters. Detailed commands are listed in Additional file 2: Section S8.

### Variant calling – QC and sample selection

Among the 235 samples, 50 exhibited a high number of SNVs. The dataset was tested for the confounding variables of sequencing depth and batch effect, and all 50 samples were in the batches sequenced on days 29-31 and days 41–43. These 50 samples were removed from further analysis, and SNVs were recalled using the SNPTools for a new consensus call set. The filtered dataset with 185 samples is presented in Table 1.

### Data merging

1. SNPTools and GATK call sets: SNVs with MAF <10% were filtered out from both call sets. A consensus site list was generated for sites that are present in both call sets, and the phased SNPtools calls for the consensus sites were used for further analysis and annotation.
2. SAS-AP and the 1000 Genomes call set: For PCA and F_ST_ analysis, samples from the 1000 Genomes Phase 3 dataset were merged with SAS-AP samples. Twenty samples were randomly chosen from each population from the 1000GP3 dataset except the SAS populations, for a total of 140, 80, 100 and 100 samples in 1000GP3-AFR, 1000GP3-AMR, 1000GP3-EAS and 1000GP3-EUR groups, respectively. All 489 samples in the 1000GP3-SAS dataset were used for the population genetic analyses. CombineVariants in GATK (version 2.4-9, [25]) was used for merging the datasets. Two different merging datasets (SAS-AP + 1000GP3-SAS, and SAS-AP + all 1000GP3 groups) were generated for different analyses.

### Population structure analysis

The smartpca module of EIGENSTRAT (version 5.0.1) [55] was used for PCA and was executed without outlier filtering. Given the novel population cohort, no linkage disequilibrium-based filtering or preprocessing was carried out. VCFtools (v0.1.12) [56] was used for calculating the mean weighted Weir-Cockerham F_ST_ between populations.

Genome-wide admixture estimates were obtained using a model-based algorithm implemented in *ADMIXTURE* (version 1.02) [28]. To eliminate the effects of SNVs that are in linkage disequilibrium, the dataset was first filtered to remove SNVs that have a pairwise r^2^ > 0.2 within 50 SNV windows using PLINK [57] as recommended by the authors of *ADMIXTURE*. Multiple *ADMIXTURE* runs were performed to cover the number of ancestral populations (K) values from 4 to 7.

### Imputation experiment

For this experiment, 6, 23, 20 and 12 samples were randomly chosen from 16 upper caste, 69 middle caste, 62 lower caste, and 38 tribal samples, respectively. This leaves 179, 162, 165 and 173 samples remaining in the SAS-AP dataset as the reference panel for imputation experiment of upper caste, middle caste, lower caste, and tribal populations, respectively. One hundred and sixty 1000GP3-SAS samples were chosen randomly from the 489 1000GP3-SAS samples and SNVs with MAF <10% were filtered out to generate a generic reference panel. The target missing SNVs were selected using a 2-stage process. First a site level intersection of all three datasets used in an imputation experiment (target, population specific and generic) was produced. Every 20^th^ site from this intersection was then removed from the consensus set, thereby deleting approximately 5% of the common sites from the target dataset for imputation. This strategy of removing SNVs ensures a genome-wide assessment of imputation accuracy where there is enough haplotype structure information remaining in the target dataset to effectively impute the missing SNVs. Beagle (ver 3.09) [58] was used to impute missing sites in the target set from the reference panels with default parameters.

## Additional files

Additional file 1: Table S1. Sequencing and variant calling statistics for 185 SAS-AP samples. (XLS 50 kb)

Additional file 2: Supplementary Information. (PDF 1370 kb)

## Abbreviations

1000GP3: 1000 Genomes Phase 3
1000GP3-AFR: 1000 Genome Project Phase 3 African
1000GP3-AMR: 1000 Genome Project Phase 3 American
1000GP3-BEB: 1000 Genome Project Phase 3 Bengalis from Bangladesh
1000GP3-EAS: 1000 Genomes Project Phase 3 East Asian
1000GP3-EUR: 1000 Genomes Project Phase 3 European
1000GP3-GIH: 1000 Genome Project Phase 3 Gujaratis in Houston
1000GP3-ITU: 1000 Genome Project Phase 3 Telugu from UK
1000GP3-PJL: 1000 Genome Project Phase 3 Punjabis from Lahore
1000GP3-SAS: 1000 Genome Project Phase 3 South Asian
1000GP3-STU: 1000 Genome Project Phase 3 Sri Lankan Tamils from UK
ANI: Ancestral North Indians
ASI: Ancestral South Indians
EXL-WGS: Extremely low coverage whole genome sequencing
GATK: Genome analysis tool kit
PCA: Principal component analysis
R^2^: dosage correlation coefficient
SAS-AP: South Asian-Andhra Pradesh
SNV: Single nucleotide variants

## References

[1] Goodwin S, McPherson JD, McCombie WR (2016). Coming of age: ten years of next-generation sequencing technologies. Nat Rev Genet.

[2] Auton A, Brooks LD, Durbin RM, Garrison EP, Kang HM, Korbel JO, Marchini JL, McCarthy S, McVean GA, Abecasis GR, 1000 Genomes Project Consortium (2015). A global reference for human genetic variation. Nature.

[3] Psaty BM, O’Donnell CJ, Gudnason V, Lunetta KL, Folsom AR, Rotter JI, Uitterlinden AG, Harris TB, Witteman JCM, Boerwinkle E (2009). Cohorts for heart and aging research in genomic epidemiology (CHARGE) consortium design of prospective meta-analyses of genome-wide association studies from 5 cohorts. Circ Cardiovasc Genet.

[4] Li Y, Sidore C, Kang HM, Boehnke M, Abecasis GR (2011). Low-coverage sequencing: implications for design of complex trait association studies. Genome Res.

[5] Fumagalli M, Vieira FG, Korneliussen TS, Linderoth T, Huerta-Sanchez E, Albrechtsen A, Nielsen R (2013). Quantifying population genetic differentiation from next-generation sequencing data. Genetics.

[6] Skotte L, Korneliussen TS, Albrechtsen A (2013). Estimating individual admixture proportions from next generation sequencing data. Genetics.

[7] Korneliussen TS, Albrechtsen A, Nielsen R (2014). ANGSD: Analysis of Next Generation Sequencing Data. BMC Bioinformatics.

[8] Nicod J, Davies RW, Cai N, Hassett C, Goodstadt L, Cosgrove C, Yee BK, Lionikaite V, McIntyre RE, Remme CA (2016). Genome-wide association of multiple complex traits in outbred mice by ultra-low-coverage sequencing. Nat Genet.

[9] Cai N, Bigdeli TB, Kretzschmar W, Li Y, Liang J, Song L, Hu J, Li Q, Jin W, Hu Z (2015). Sparse whole-genome sequencing identifies two loci for major depressive disorder. Nature.

[10] Pasaniuc B, Rohland N, McLaren PJ, Garimella K, Zaitlen N, Li H, Gupta N, Neale BM, Daly MJ, Sklar P (2012). Extremely low-coverage sequencing and imputation increases power for genome-wide association studies. Nat Genet.

[11] Adzhubei IA, Schmidt S, Peshkin L, Ramensky VE, Gerasimova A, Bork P, Kondrashov AS, Sunyaev SR (2010). A method and server for predicting damaging missense mutations. Nat Methods.

[12] Gibbs RA, Belmont JW, Hardenbol P, Willis TD, Yu F, Yang H, Ch’ang L-Y, Huang W, Liu B, Shen Y (2003). The international HapMap project. Nature.

[13] International HapMap Consortium (2010). Integrating common and rare genetic variation in diverse human populations. Nature.

[14] Singh KS (2002). People of India: an introduction.

[15] Chaubey G, Metspalu M, Kivisild T, Villems R (2007). Peopling of South Asia: investigating the caste–tribe continuum in India. Bioessays.

[16] Reich D, Thangaraj K, Patterson N, Price AL, Singh L (2009). Reconstructing Indian population history. Nature.

[17] Basu A, Sarkar-Roy N, Majumder PP (2016). Genomic reconstruction of the history of extant populations of India reveals five distinct ancestral components and a complex structure. Proc Natl Acad Sci.

[18] Xing J, Watkins WS, Hu Y, Huff CD, Sabo A, Muzny DM, Bamshad MJ, Gibbs RA, Jorde LB, Yu F (2010). Genetic diversity in India and the inference of Eurasian population expansion. Genome Biol.

[19] Simonson TS, Zhang Y, Huff CD, Xing J, Watkins WS, Witherspoon DJ, Woodward SR, Jorde LB (2010). Limited distribution of a cardiomyopathy-associated variant in India. Ann Hum Genet.

[20] Wong L-P, Lai JK-H, Saw W-Y, Ong RT-H, Cheng AY, Pillai NE, Liu X, Xu W, Chen P, Foo J-N (2014). Insights into the genetic structure and diversity of 38 South Asian Indians from deep whole-genome sequencing. PLoS Genet.

[21] Clark AG, Hubisz MJ, Bustamante CD, Williamson SH, Nielsen R (2005). Ascertainment bias in studies of human genome-wide polymorphism. Genome Res.

[22] Lachance J, Tishkoff SA (2013). SNP ascertainment bias in population genetic analyses: why it is important, and how to correct it. Bioessays.

[23] Lander ES, Waterman MS (1988). Genomic mapping by fingerprinting random clones: a mathematical analysis. Genomics.

[24] Wang Y, Lu J, Yu J, Gibbs RA, Yu F (2013). An integrative variant analysis pipeline for accurate genotype/haplotype inference in population NGS data. Genome Res.

[25] McKenna A, Hanna M, Banks E, Sivachenko A, Cibulskis K, Kernytsky A, Garimella K, Altshuler D, Gabriel S, Daly M (2010). The genome analysis toolkit: a MapReduce framework for analyzing next-generation DNA sequencing data. Genome Res.

[26] Xing J, Watkins WS, Witherspoon DJ, Zhang Y, Guthery SL, Thara R, Mowry BJ, Bulayeva K, Weiss RB, Jorde LB (2009). Fine-scaled human genetic structure revealed by SNP microarrays. Genome Res.

[27] Bamshad M, Kivisild T, Watkins WS, Dixon ME, Ricker CE, Rao BB, Naidu JM, Prasad BV, Reddy PG, Rasanayagam A (2001). Genetic evidence on the origins of Indian caste populations. Genome Res.

[28] Alexander DH, Novembre J, Lange K (2009). Fast model-based estimation of ancestry in unrelated individuals. Genome Res.

[29] Neuvonen AM, Putkonen M, Översti S, Sundell T, Onkamo P, Sajantila A, Palo JU (2015). Vestiges of an ancient border in the contemporary genetic diversity of north-eastern Europe. PLoS One.

[30] Norio R. Genetics and the Origin of the Finns. eLS. 2013. doi:10.1002/9780470015902.a0020806.pub2.

[31] Watkins WS, Thara R, Mowry BJ, Zhang Y, Witherspoon DJ, Tolpinrud W, Bamshad MJ, Tirupati S, Padmavati R, Smith H (2008). Genetic variation in South Indian castes: evidence from Y-chromosome, mitochondrial, and autosomal polymorphisms. BMC Genet.

[32] Indian Genome Variation Consortium (2008). Genetic landscape of the people of India: a canvas for disease gene exploration. J Genet.

[33] Marchini J, Cardon LR, Phillips MS, Donnelly P (2004). The effects of human population structure on large genetic association studies. Nat Genet.

[34] Freedman ML, Reich D, Penney KL, McDonald GJ, Mignault AA, Patterson N, Gabriel SB, Topol EJ, Smoller JW, Pato CN (2004). Assessing the impact of population stratification on genetic association studies. Nat Genet.

[35] Maloney C (1974). The races in peoples of South Asia.

[36] Chandler WB (1985). The Ethiopian presence in the Indus valley civilization. J Afr Civilizations.

[37] Cavalli-Sforza LL, Menozzi P, Piazza A. The history and geography of human genes. Princeton: Princeton university press; 1994.

[38] Browning SR, Browning BL (2007). Rapid and accurate haplotype phasing and missing-data inference for whole-genome association studies by use of localized haplotype clustering. Am J Hum Genet.

[39] Davies RW, Flint J, Myers S, Mott R (2016). Rapid genotype imputation from sequence without reference panels. Nat Genet.

[40] Pemberton TJ, Jakobsson M, Conrad DF, Coop G, Wall JD, Pritchard JK, Patel PI, Rosenberg NA (2008). Using population mixtures to optimize the utility of genomic databases: linkage disequilibrium and association study design in India. Ann Hum Genet.

[41] Guo Y, Long J, He J, Li CI, Cai Q, Shu XO, Zheng W, Li C. Exome sequencing generates high quality data in non-target regions. BMC genomics. 2012;13(1):194. 10.1186/1471-2164-13-194 PMC3416685 22607156

[42] Cancer Genome Atlas Research N (2013). Genomic and epigenomic landscapes of adult de novo acute myeloid leukemia. N Engl J Med.

[43] Kivisild T, Bamshad MJ, Kaldma K, Metspalu M, Metspalu E, Reidla M, Laos S, Parik J, Watkins WS, Dixon ME (1999). Deep common ancestry of Indian and western-Eurasian mitochondrial DNA lineages. Curr Biol.

[44] Richards M, Macaulay V, Hickey E, Vega E, Sykes B, Guida V, Rengo C, Sellitto D, Cruciani F, Kivisild T (2000). Tracing European founder lineages in the Near Eastern mtDNA pool. Am J Hum Genet.

[45] Illumina - AllSeq. [http://allseq.com/knowledge-bank/sequencing-platforms/illumina/]. Accessed 20 June 2016.

[46] Rohland N, Reich D (2012). Cost-effective, high-throughput DNA sequencing libraries for multiplexed target capture. Genome Res.

[47] Wang C, Zhan X, Bragg-Gresham J, Kang HM, Stambolian D, Chew EY, Branham KE, Heckenlively J, Study TF, Fulton R (2014). Ancestry estimation and control of population stratification for sequence-based association studies. Nat Genet.

[48] Bamshad MJ, Watkins WS, Dixon ME, Jorde LB, Rao BB, Naidu JM, Prasad BVR, Rasanayagam A, Hammer MF (1998). Female gene flow stratifies Hindu castes. Nature.

[49] Watkins WS, Bamshad M, Dixon ME, Rao BB, Naidu JM, Reddy PG, Prasad B, Das PK, Reddy PC, Gai PB (1999). Multiple origins of the mtDNA 9-bp deletion in populations of South India. Am J Phys Anthropol.

[50] BCM-HGSC [https://www.hgsc.bcm.edu/sites/default/files/documents/Illumina_Barcoded_Paired-End_Capture_Library_Preparation.pdf]. Accessed 20 June 2016.

[51] Li H, Durbin R (2010). Fast and accurate long-read alignment with Burrows–Wheeler transform. Bioinformatics.

[52] Reid JG, Carroll A, Veeraraghavan N, Dahdouli M, Sundquist A, English A, Bainbridge M, White S, Salerno W, Buhay C (2014). Launching genomics into the cloud: deployment of Mercury, a next generation sequence analysis pipeline. BMC bioinformatics.

[53] The 1000 Genomes Project Consortium (2012). An integrated map of genetic variation from 1,092 human genomes. Nature.

[54] GATK | Index [https://www.broadinstitute.org/gatk/guide/best-practices.php]. Accessed 20 June 2013.

[55] Patterson N, Price AL, Reich D (2006). Population structure and eigenanalysis. PLoS Genet.

[56] Danecek P, Auton A, Abecasis G, Albers CA, Banks E, DePristo MA, Handsaker RE, Lunter G, Marth GT, Sherry ST (2011). The variant call format and VCFtools. Bioinformatics.

[57] Purcell S, Neale B, Todd-Brown K, Thomas L, Ferreira MAR, Bender D, Maller J, Sklar P, De Bakker PIW, Daly MJ (2007). PLINK: a tool set for whole-genome association and population-based linkage analyses. Am J Hum Genet.

[58] Browning BL, Browning SR (2016). Genotype imputation with millions of reference samples. Am J Hum Genet.

