# Supplementary material for: Extremely low-coverage whole genome sequencing in South Asians captures population genomics information
Source: BMC Genomics. 2017 May 22;18:396. doi: 10.1186/s12864-017-3767-6 (PMC5440948; doi:10.1186/s12864-017-3767-6)
Supplement: Supplementary file 2 — Supplementary Information. (PDF 1370 kb) [file 12864_2017_3767_MOESM2_ESM.pdf]

Supplementary Material

for

Extremely low-coverage whole genome sequencing in South Asians captures population genomics information

Navin Rustagi, Anbo Zhou, W. Scott Watkins, Erika Gedvilaite, Shuoguo Wang, Naveen Ramesh, Donna Muzny, Richard A Gibbs, Lynn B Jorde*, Fuli Yu*, and Jinchuan Xing*

Table of Contents

S1. Lander Waterman statistics 2
S2. Simulation experiment for SNV calling of EXL-WGS 2
S2.1 Optimizing SNPTools parameters for variant calling 2
S2.2 Variant calling results from the simulation experiment 4
S3. Assessing the quality of SNPTools and GATK call sets 7
S4. Variant site coverage and heterozygous genotype call quality. 9
S4.1 Average variant site coverage in the final dataset. 9
S4.2 Heterozygous genotype calls quality in the final dataset. 10
S5 Population genetics analysis 11
S5.1 Additional genotype-based analyses. 11
S5.2 Genotype likelihood based analyses. 14
S6. Simulation experiments for imputation analysis 18
S7. Evaluation of different Indian reference panels for imputation analysis 19
S8. Commands for GATK pipeline for the SAS-AP data 22

S1. Lander Waterman statistics

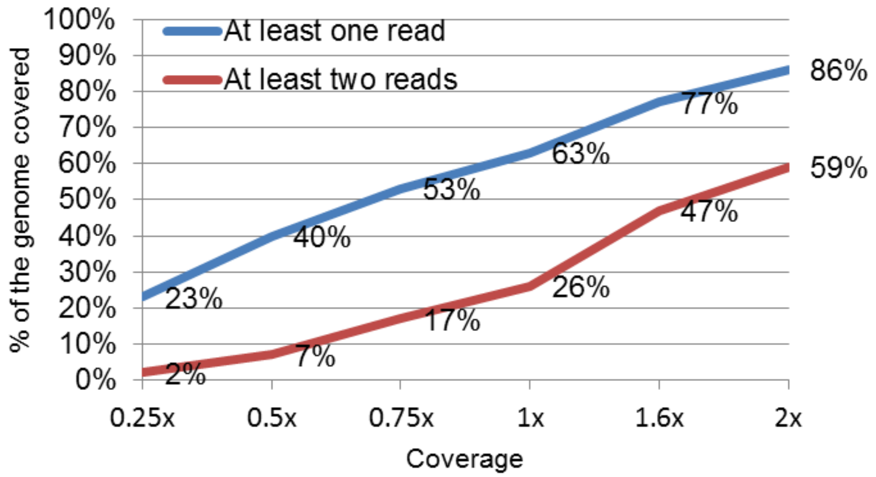

Figure S1.1: Percentage of the genomes covered for different average coverages predicted by Lander Waterman statistics [1].

S2. Simulation experiment for SNV calling of EXL-WGS

S2.1 Optimizing SNPTools parameters for variant calling

The variance ratio statistic parameter (*s)* of the SNPTools pipeline computes the ratio between extra binomial variation and best genotype fit and is computed from aggregated data across all samples for a particular site. Increasing the value of *s* allows for more stringent filtering of false positive variants, while also decreasing the sensitivity.

Protocol 1: Down-sample bam with coverage C to coverage x, where x < C

1. Require: Bam File, Float x
2. Ensure: Bam File with coverage >x
3. N<- (x *3,000,000, 000)/(read length)
4. T <- read ids which have both reads mapped.
5. T' <-Uniform sample of N/2 read ids from T
6. Output bam with only read ids in T' using Picard Tools

In this section we present evidence for *s=2.8* being the ideal value for the SNPTools pipeline for our data. All the analyses are for chromosome 20. For this analysis, we generated a simulated cohort of samples consisting of down-sampled 1000Genomes

African (AFR) WGS data in BAM format. Protocol 1 was used to down-sample BAMs. The input coverages mimicked that of the SAS-AP dataset. Only AFR samples were considered for this analysis, because African populations are the most diverse and contain the highest number of SNVs among all the populations in 1000 Genomes project. We refer to the down-sampled BAMs from the 1000 Genomes project African samples as SAFR. To ensure quality and consistency of our down-sampled BAMs, we only choose the 208 samples which were sequenced with the Illumina GAII platform and above. In Figure S2.1A, we present transition/transversion (Ti/Tv) ratios for novel SNVs and shared SNVs in SAFR for s = 1.8, 2.0, 2.5, 2.8 with respect to the SNV's released as part of the 1000 Genomes consortium [2]. The Ti/Tv ratio for chromosome 20 is 2.37 in the 1000 Genomes phase 1 dataset but the Ti/Tv ratio for novel SNVs at s=2.8 is 1.02. Since the total number of transversions is twice as that of transitions, the Ti/Tv ratio of 1.08 is likely because of random errors and not because of other biological properties of the human genome. The number of potential false positive SNVs for s = 2.8 is 8,910 (3%), which is well within bounds predicted by sequencing error rates for the Illumina platform. While increasing values of s reduced the true positive rate, it also reduces the false positive rate to within bounds expected due to sequencing errors at this coverage (Figure S2.1B). The false positive rate is expected to saturate at this point.

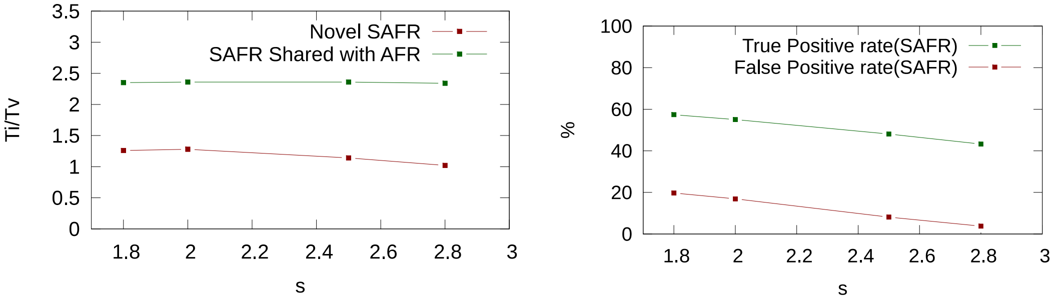

Figure S2.1: Effect of the variance ratio statistic parameter (*s*) on variant discovery. A) Effect of *s* on the Ti/Tv ratio. Novel SAFR sites are ones that are in SAFR but not in AFR. The shared sites are present in both. **B) Effect of *s* on the variant calling quality.** The false positive rate is defined as total number of novel SNVs divided by the total number of SNVs in the experimental set. The true positive rate is defined as the total number of shared SNVs by the total number of SNVs in the control set. Both plots are for chromosome 20.

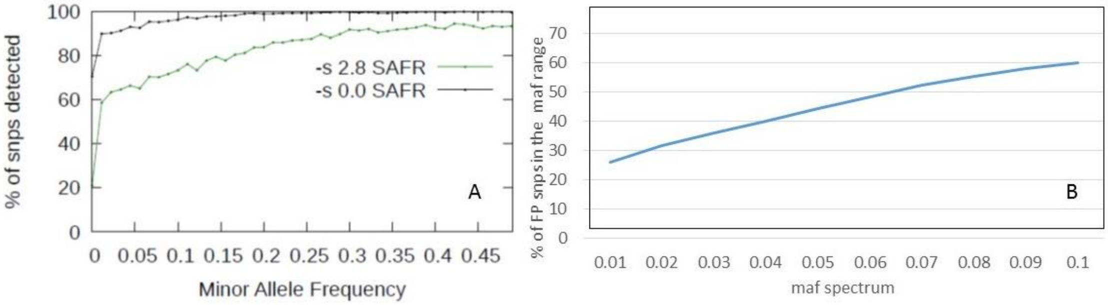

Figure S2.2: A) The recovery of SNVs with respect to the minor allele frequency (MAF) spectrum. The MAF spectrum is divided into 45 bins. The value of *s* = 0 gives an estimate on the number of SNVs missed due to lack of coverage. For MAF ≥ 10%, over 65% of SNVs are recovered at s = 2.8. **B) Cumulative distribution of false positive SNVs across the MAF spectrum.** All results are for chromosome 20 on the SAFR dataset.

S2.2 Variant calling results from the simulation experiment

Using the down-sampled SAFR dataset, we evaluated the variant calling performance of SNPTools. Phased and imputed SAFR dataset was generated using SNPTools. In figure S2.2A we present the distribution of SNV rediscovery rate across the minor allele frequency (MAF) spectrum for the SAFR dataset. For MAF = 10% close to 65% of the SNVs are recalled in the same MAF category and the recall-rate increases to >80% for MAF ≥ 20%. The cumulative distribution of false positive SNVs across the MAF spectrum shows 60% of false positive SNVs have MAF < 10% (Figure S2.2B). The average individual genotype discordance rate between the SAFR SNPTools output and the 1000 Genomes AFR release is 5.01% (± 2.83%). Only 11/208 samples have higher than 7% discordance rate. For the dataset containing sites with MAF ≥ 10%, the average individual discordance is 6.43% (±4.93%). Only 43 SNVs disagree with the 1000 Genomes AFR dataset on alternate alleles.

To estimate the effect of discordance rate on population substructure, we performed PCA on simulated cohorts with 0.25x, 0.5x, 0.75x and 1x average coverage for 500 samples across the 1000 Genomes Phase 1 populations using sites with MAF ≥ 20%. The 500 samples include 100,175,125 and 100 samples randomly selected from AFR, EUR, EAS and AMR populations, respectively. Even for coverages as low as 0.25x, PCA on sites with MAF ≥20% is sufficient to detect population structure among the four main groups (Figure S2.3).

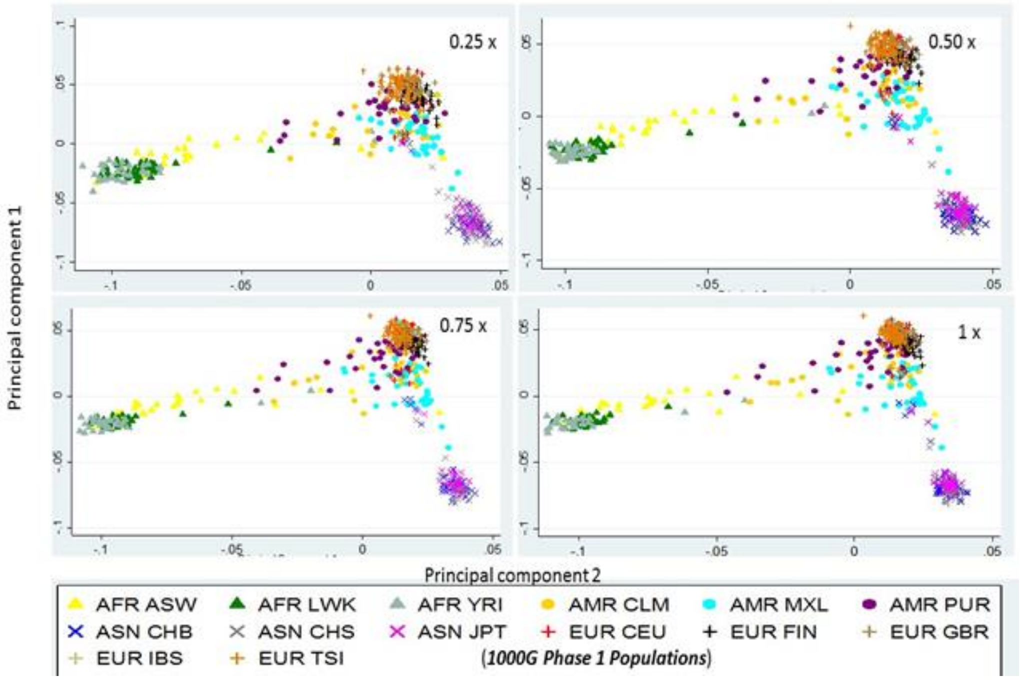

Figure S2.3: PCA of simulated cohort of 500 samples from the 1000 Genomes Phase 1 with coverage 0.25x, 0.50x, 0.75x and 1x and MAF ≥20%. Eigenstrat was used to generate the PCA plots.

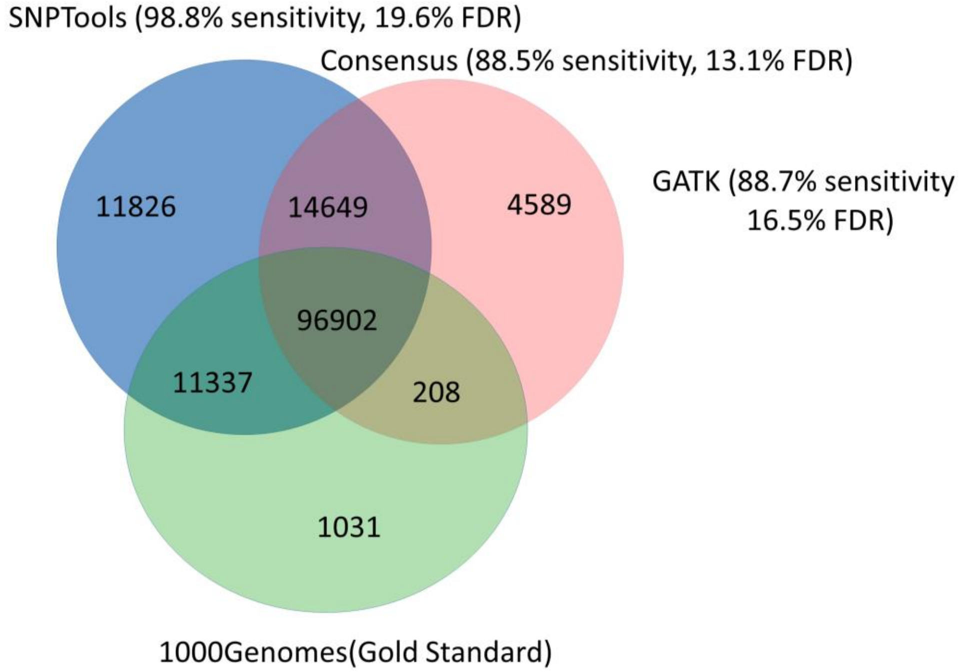

Figure S2.4: Calling sensitivity and FDR for SNPTools and GATK. The comparison was for chromosome 20. All the datasets contain SNVs with MAF≥10%.

S3. Assessing the quality of SNPTools and GATK call sets

To assess the quality of the EXL-WGS call set, we compared our EXL-WGS call set in an ENCODE region (chr12: 40,540,210 - 40,640,209) with a previously published results based on Sanger sequencing (referred as ENCODE dataset hereafter) [3]. Because the ENCODE dataset was subjected to a stringent quality filtering procedure, it should be considered as a high-quality variant call set instead of a full representation of all variants in this region. A total of 63 individuals overlap between the two studies and 402 SNVs were reported in the ENCODE study. In this region GATK called 508 SNVs with 276 overlap with ENCODE data set; while SNPTools called 358 SNVs and 265 sites overlap with ENCODE data set. The GATK and SNPTools intersection has 353 sites, and 263 sites (74.5%) overlap with ENCODE data set.

To further examine the difference between the call sets, we determined the overlap among datasets at different MAF categories. As expected, the majority of false-negative sites in GATK and SNPTools call sets are rare: 92.1% (128/139) of the ENCODE sites that are not called by GATK and SNPTools intersection have MAF < 10% (Figure S3.1). Comparing with the individual GATK and SNPTools call sets, the intersection set has a comparable number of overlapping sites in each MAF categories (Figure S3.1). In addition, MAFs of SNVs in the intersection set are in high correlation with MAFs of SNVs in the ENCODE dataset with an r^2 value of 0.987 (Figure S3.2).

With MAF ≥ 10% site, 203 SNVs remain in the ENCODE dataset. GATK called 272 SNVs with 185 overlap with ENCODE dataset; while SNPTools called 252 SNVs and 190 sites overlap with ENCODE data set. The GATK and SNPTools intersection has 235 sites, and 177 of them (75.3%) overlap with ENCODE data set.

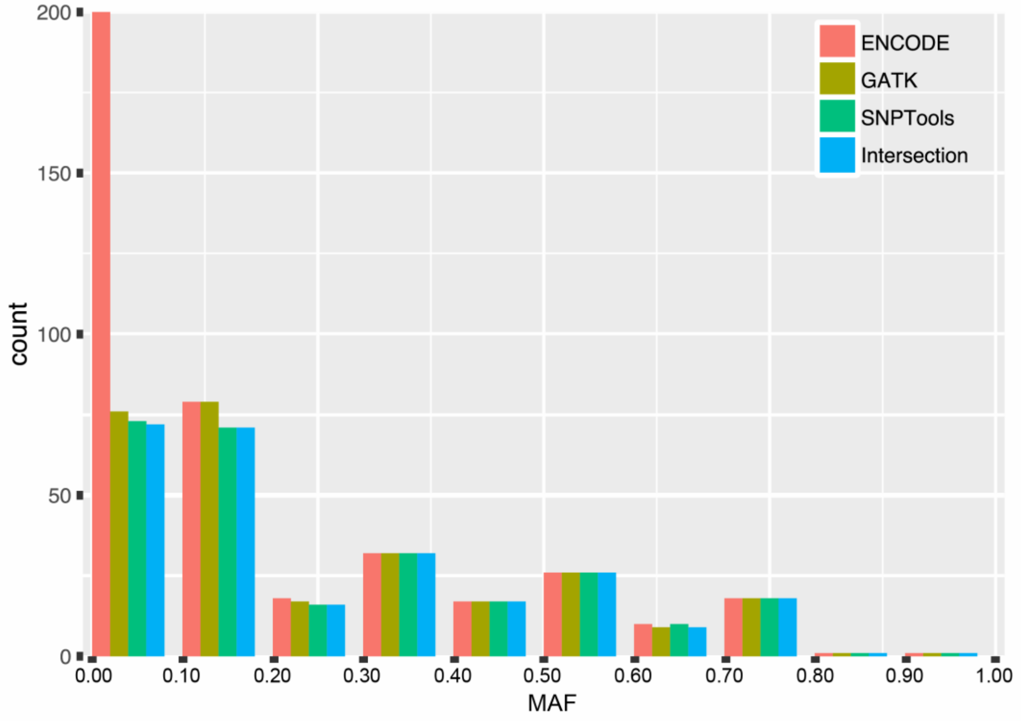

Figure S3.1: The number of variant site in different MAF categories. MAF is calculated using the ENCODE data. The number of GATK, SNPTools, and SNPTools-GATK intersection calls that overlap ENCODE data in each MAF categories are shown.

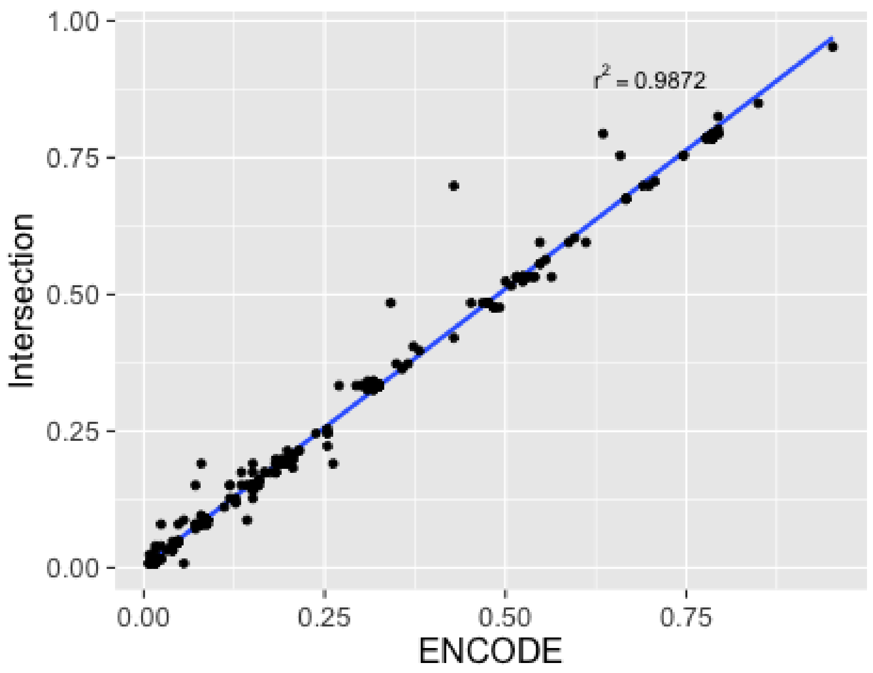

Figure S3.2: Allele frequency correlation between the ENCODE dataset and the EXL-WGS dataset.

S4. Variant site coverage and heterozygous genotype call quality.

S4.1 Average variant site coverage in the final dataset.

The sequencing coverage of called SNVs in the release call set is shown in Figure S4.1. As is expected based on our average coverage of 1.6x, the vast majority of the SNV sites have an average coverage between 0.5x to 4x. There are 428 SNVs with average coverage 10x or more. There are 21,249 SNVs with average coverage 0.5x or less. The lowest average coverage over 185 samples for an SNV is ~0.03x.

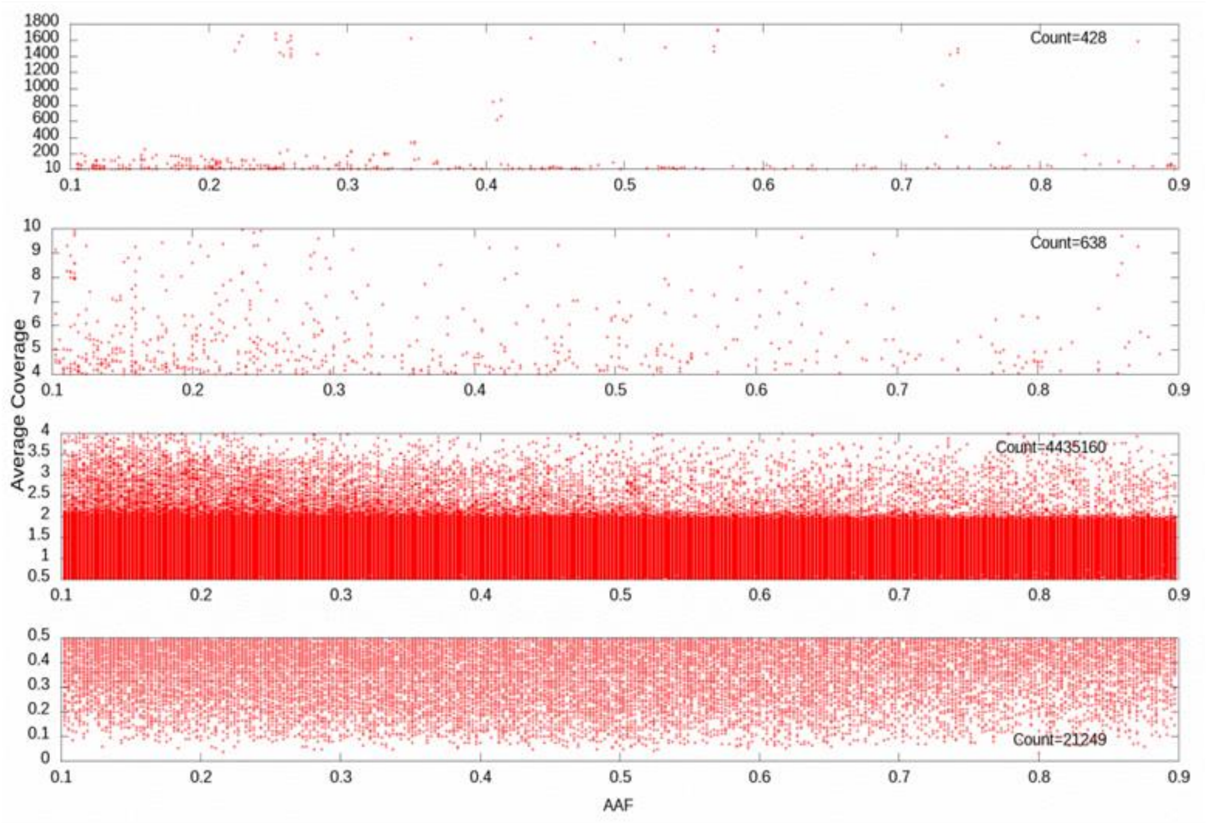

Figure S4.1: A four panel scatter plot for alternate allele frequency (AAF, x-axis) versus average coverage for 185 samples (y-axis). The count of SNV's in each y-axis range in the panel is presented within in the panel.

S4.2 Heterozygous genotype calls quality in the final dataset.

The final SAS-AP dataset goes through joint calling followed by phasing and imputation using SNPTools. We expect to achieve high calling accuracy for heterozygous SNVs in our consensus call set. To explore the confidence in calling heterozygous SNVs, we compared the calls with the gold standard ENCODE dataset. For the 175 sites that are called by both ENCODE and the SAS-AP dataset with consistent alternate alleles, the total number of genotypes for the 63 shared samples are 175*63=11,025. Of these, 10,953 were genotyped in the ENCODE data set. Among the 10,953 genotypes 4,301 were called as heterozygous, with 4,231 (98.4%) correctly called by EXL-WGS (Supplemental figure S4.2). We achieved high genotyping accuracy (97.3%) even for heterozygous sites that have no coverage in the individual. This result highlights the power of imputation after joint genotype calling.

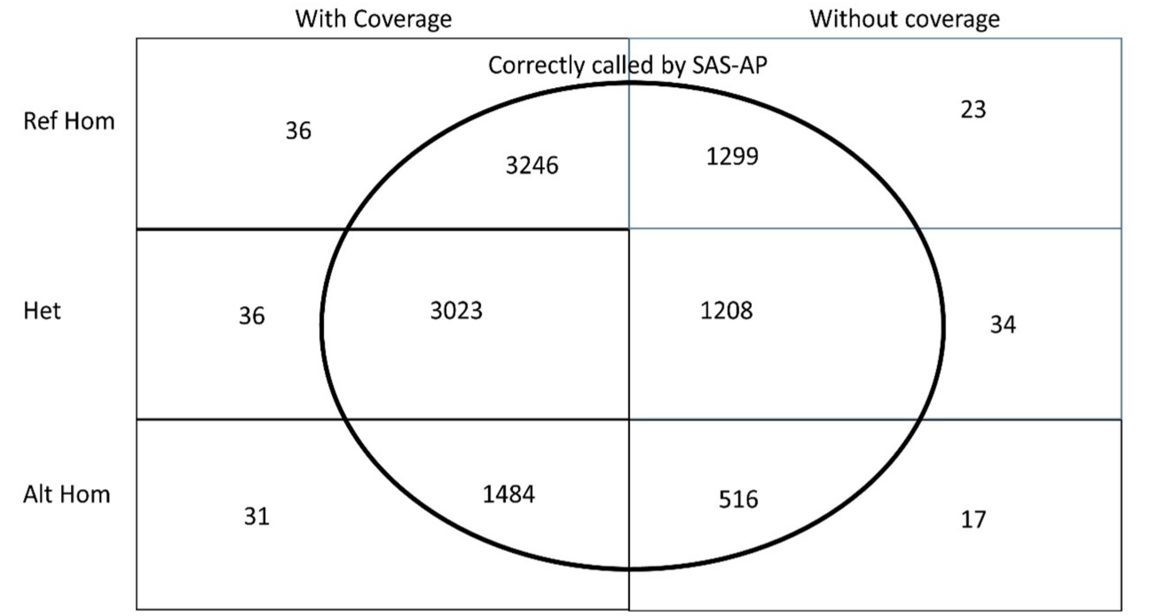

Figure S4.2: Genotype comparison between ENCODE dataset and EXL-WGS calls.

S5 Population genetics analysis

S5.1 Additional genotype-based analyses.

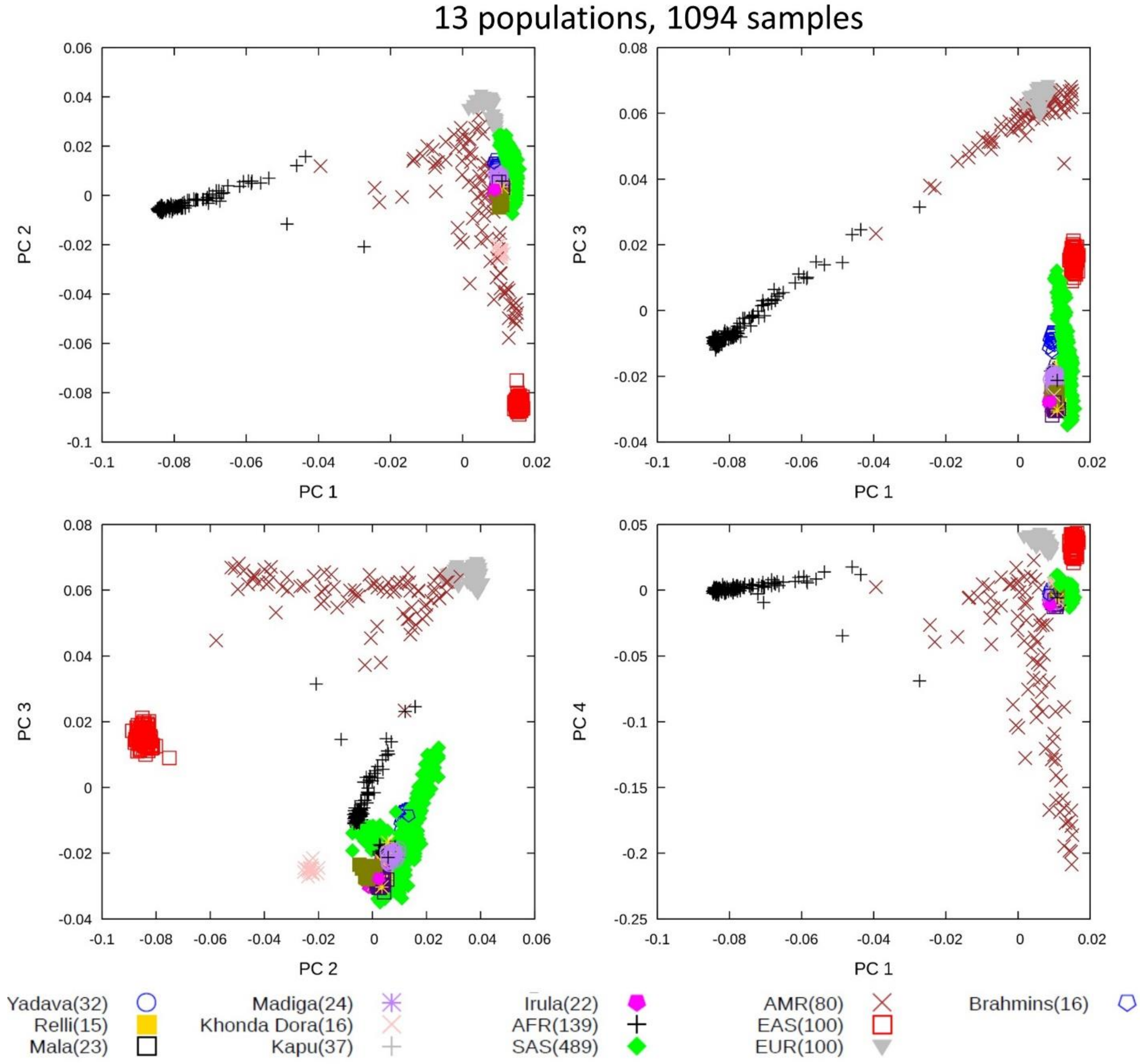

Figure S5.1: PCA of SAS-AP and 1000GP3 samples. Different combination of PC1 to PC4 are shown in subpanels.

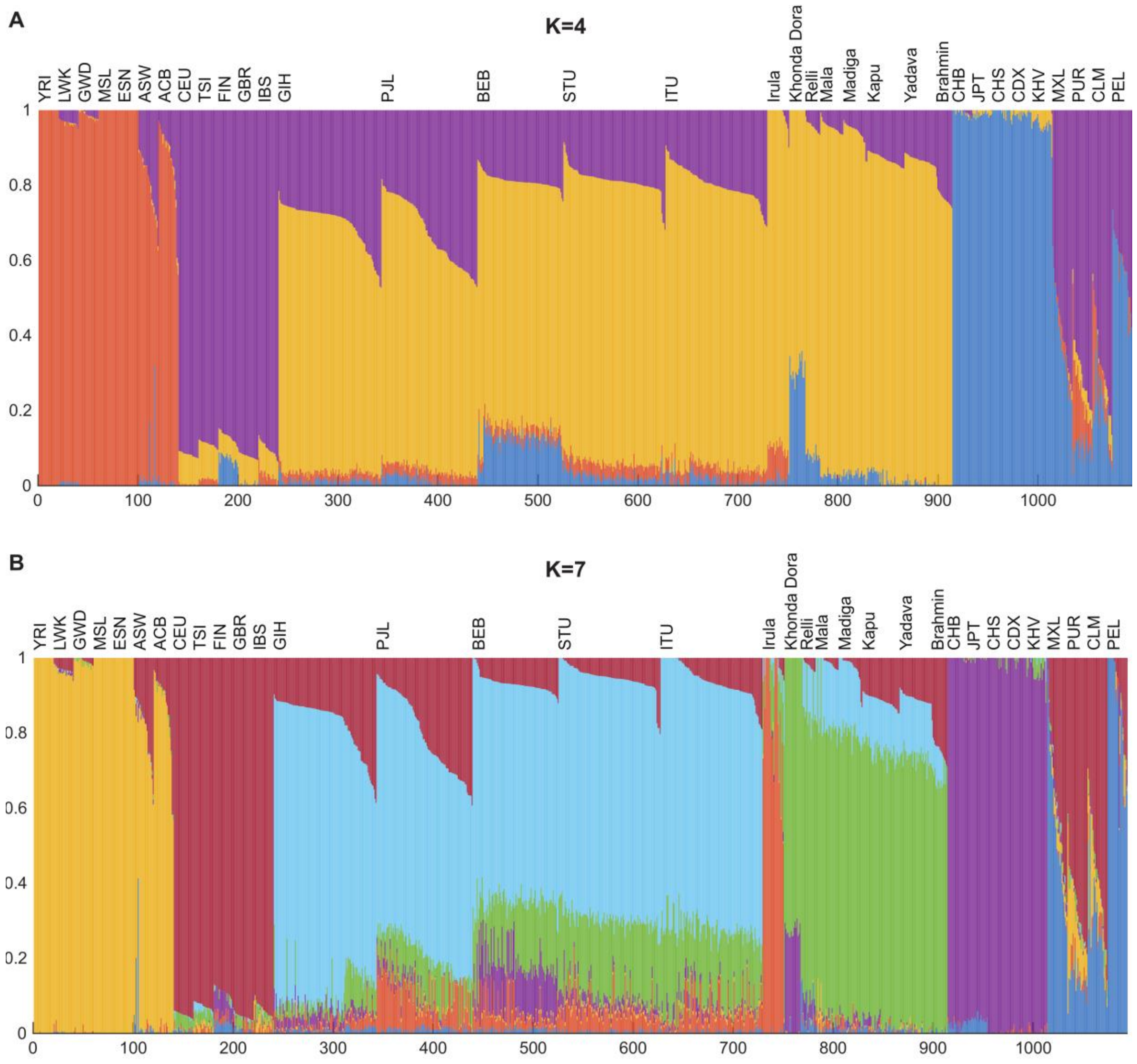

Figure S5.2: Admixture plot for SAS-AP with 909 1000GP3 samples. A) K = 4; B) K = 7. Each vertical bar represents one sample. The vertical bar is composed of colored sections, where each section represents the proportion of a sample's ancestry derived from one of K ancestral populations.

Table S5.1 Weighted F_{ST} between SAS-AP and 1000GP3 populations.

	SAS	EUR	EAS	AMR	AFR
**Kapu**	0.002	0.043	0.068	0.038	0.122
**Brahmins**	0.002	0.035	0.076	0.034	0.124
**Relli**	0.008	0.057	0.067	0.047	0.131
**Mala**	0.005	0.055	0.071	0.047	0.128
**Madiga**	0.004	0.053	0.069	0.045	0.128
**Irula**	0.027	0.074	0.089	0.065	0.144
**Yadava**	0.003	0.045	0.072	0.041	0.124
**Khonda Dora**	0.028	0.082	0.055	0.061	0.144

Table S5.2 Weighted F_{ST} between SAS-AP populations.

	Brahmin	Irula	Kapu	Khonda Dora	Madiga	Mala	Relli
**Brahmin**	-						
**Irula**	0.0305						
**Kapu**	0.0022	0.0263					
**Khonda Dora**	0.033	0.0455	0.0245				
**Madiga**	0.0059	0.0261	0.0021	0.0228			
**Mala**	0.0069	0.0264	0.0023	0.0228	0.0008		
**Relli**	0.0104	0.0301	0.0057	0.0224	0.0052	0.0053	
**Yadava**	0.0037	0.0278	0.0017	0.0273	0.0032	0.0038	0.0073

Table S5.3 Weighted F_{ST} between SAS-AP and 1000GP3-SAS populations.

	ITU	STU	BEB	PJL	GIH
**Brahmins**	0.0031	0.0036	0.0046	0.0035	0.0049
**Kapu**	0.0018	0.0021	0.0030	0.0050	0.0056
**Yadava**	0.0033	0.0033	0.0046	0.0063	0.0069
**Mala**	0.0045	0.0042	0.0051	0.0096	0.0098
**Madiga**	0.0040	0.0037	0.0045	0.0085	0.0087
**Relli**	0.0083	0.0079	0.0071	0.0125	0.0131
**Khonda Dora**	0.0284	0.0267	0.0233	0.0346	0.0350
**Irula**	0.0276	0.0266	0.0279	0.0315	0.0324

S5.2 Genotype likelihood based analyses.

Genotype likelihood (GL) based analyses were performed using the program angsd [4] and ngsTools [5].

Principal component analysis: Posterior probabilities of the three genotypes at each site in each sample were calculated using angsd. The covariance matrix was then calculated by ngsCovar [6] within ngsTools [4] using the posterior probabilities. PCA was performed on the covariance matrix.

Admixture analysis: Genotype likelihoods for each site in each sample were calculated using angsd. Admixture analysis was then performed by ngsAdmix [7] using the genotype likelihoods.

F_{ST}: the site allele frequency likelihood was calculated for each population using angsd [8]. Pairwise population F_{ST} was then calculated by realSFS within angsd using the site allele frequency likelihood in 50 Kb sliding window with 10 Kb step, as the program recommended. The mean **F_{ST}** of all windows are reported.

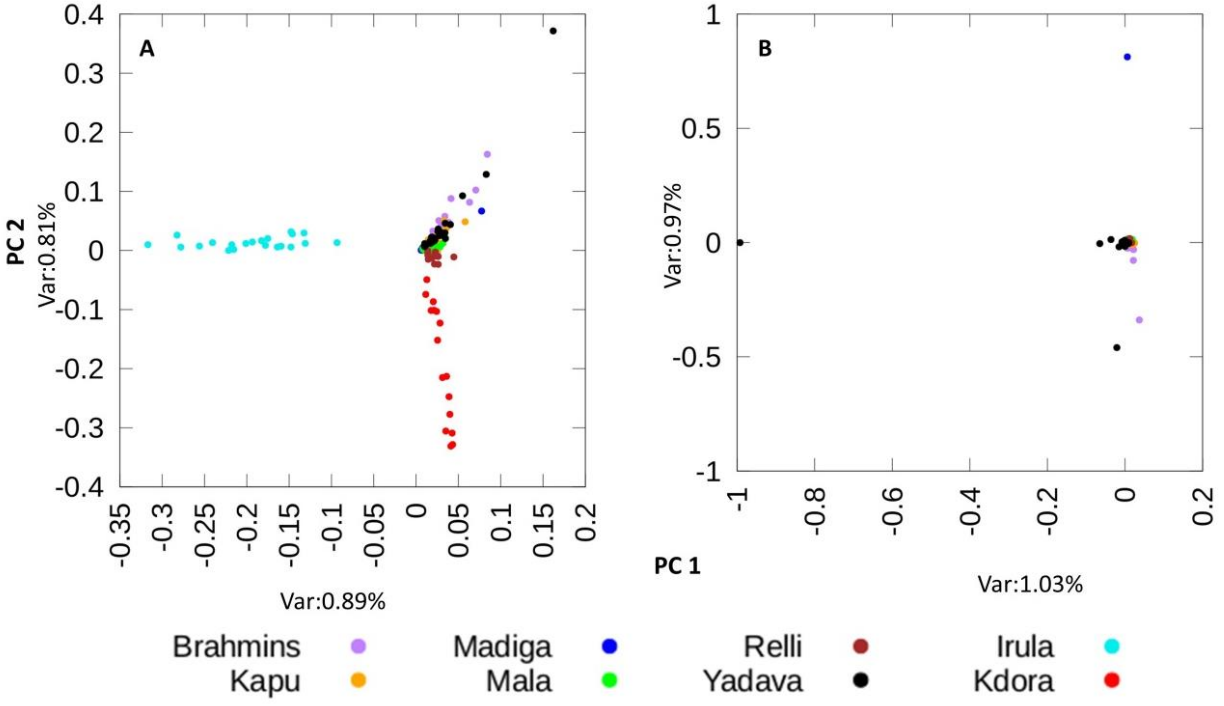

Figure S5.3: GL-based PCA of SAS-AP samples. A) All SAS-AP samples; **B)** SAS-AP excluding Khonda Dora and Irula samples. Each dot represents one individual. PC1 and PC2 are shown on the X and Y axis, respectively. The variance explained by each PC is labeled on the axis.

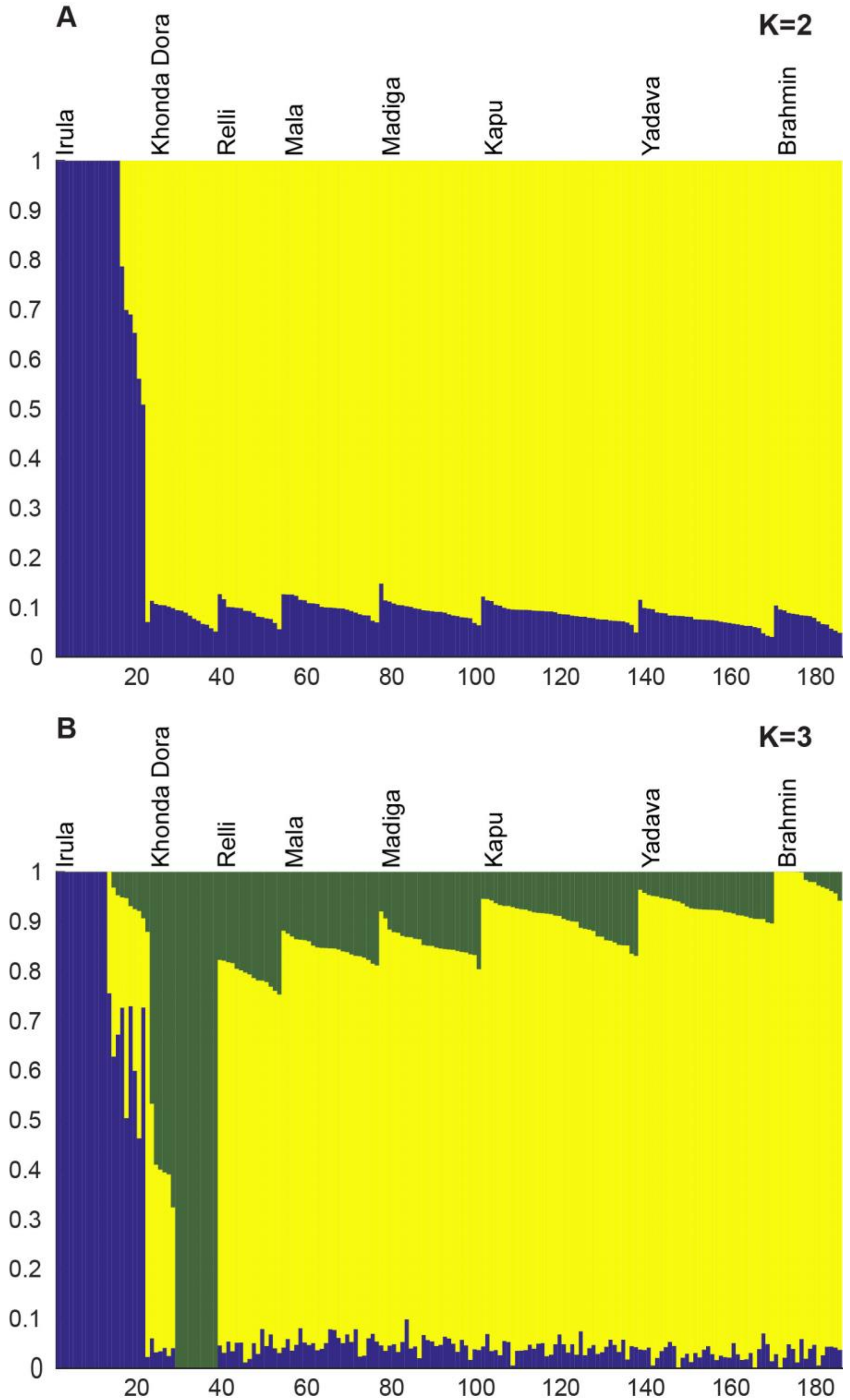

Figure S5.4: GL-based admixture plot for SAS-AP samples. A) K = 2; B) K = 3. Each vertical bar represents one sample. The vertical bar is composed of colored sections, where each section represents the proportion of a sample's ancestry derived from one of K ancestral populations.

Table S5.4 GL-based F_{ST} between SAS-AP populations (angsd).

	Brahmin	Irula	Kapu	Khonda Dora	Madiga	Mala	Relli
**Irula**	0.029						
**Kapu**	0.009	0.026					
**Khonda Dora**	0.030	0.039	0.025				
**Madiga**	0.011	0.025	0.008	0.023			
**Mala**	0.012	0.026	0.008	0.023	0.007		
**Relli**	0.014	0.028	0.011	0.022	0.010	0.010	
**Yadava**	0.010	0.028	0.007	0.028	0.009	0.009	0.013

S6. Simulation experiments for imputation analysis

To determine the feasibility of creating a reference imputation panel using EXL-WGS dataset, we compared the performance of an imputation reference panel from a simulated EXL-WGS cohort to that of a SNP array dataset using the same samples. For this comparison, we generated a dataset of 185 AFR samples as described in Protocol 1 in Supplementary Section S2 with average depth of coverage 1.6x. All the analyses were carried out on chromosome 20 for this experiment. The SNPTools pipeline was used to call variants in the dataset. Among them, 145 samples were used as the reference panel and 40 samples were used as the target set for imputation. The Affymetrix SNP array dataset was downloaded from (http://www.1000genomes.org/category/high-density-genotyping/) and filtered to remove SNVs with MAF <10%. The final SNP array dataset contains a total of 15,887 SNVs. The gold standard missing SNVs are selected using the 2-stage process described in methods. Approximately 5% of SNV's from the consensus of the three sets (14,125) are removed from the target set. Beagle (ver 3.09) [9] was used to impute missing sites in the target set from the reference panels with default parameters. All missing SNVs were recovered with both EXL-WGS and SNP array reference panels with an average individual genotype discordance rate of 6%. These results demonstrate that the EXL-WGS design can produce an imputation reference panel that is comparable with SNP array in populations that have a good coverage on the SNP array.

S7. Evaluation of different Indian reference panels for imputation analysis

We tested the imputation performance of both SAS-AP and the 1000 Genomes Indian reference panels on the 1000 Genomes Indian samples. For this analysis, we chose 23 target samples from the ITU (Indian Telugu from the UK) populations and removed 5% of the sites, using the same methodology as described in the method section. For this target set, we performed two experiments.

Experiment 1

Compare the relative performance of the following reference panels

a. 69 ITU Samples (randomly selected)
b. 69 middle caste SAS-AP samples (All Samples)
c. 69 lower caste and middle caste SAS-AP samples (randomly selected)
d. 62 lower caste SAS-AP samples (All)
e. 69 non-tribal SAS-AP samples (Randomly selected)

The reference panels of a) and b) were limited to the consensus sites between a) and b). All the other reference panels had less number of sites than a) and b).

Experiment 2

Compare two reference panels constructed from the 1000 Genomes South Asian (SAS) samples to the entire SAS-AP dataset for the 23 target ITU samples. The 1000 Genomes SAS samples include samples from five populations: Gujarati Indian from Houston, Texas (GIH), Punjabi from Lahore, Pakistan (PJL), Bengali from Bangladesh (BEB), Sri Lankan Tamil from the UK (STU), and Indian Telugu from the UK (ITU). The two reference panels were made by randomly choosing:

a. 185 1000 Genomes SAS samples excluding ITU samples

b. 185 1000 Genomes SAS samples excluding ITU and STU samples.

All the reference panels compared in this experiment were limited to the sites which lie in the consensus of the reference panels.

Results of the mean Dosage R^2 statistics for each of the reference panels in imputing the missing SNVs from the 23 target ITU samples in experiment 1 are presented in Figure S7.1. The ITU reference panel has the best overall performance, as expected. Among SAS-AP reference panels, samples from all non-tribal population have the best performance. This experiment shows that distance from the target populations plays an important role in imputation accuracy.

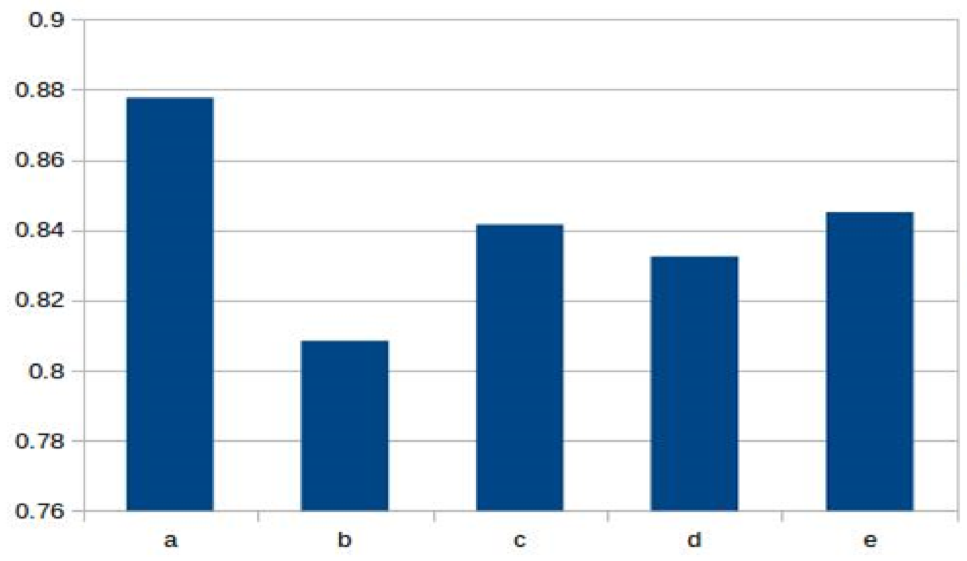

Figure S7.1: Mean Dosage R^2 of various imputation reference panels for a target set of 23 ITU samples in experiment 1.

Results of mean Dosage R^2 for experiment 2 are shown in figure S7.2. The reference panel including the STU samples has a significant gain in imputation accuracy over the SAS-AP dataset, but this difference cannot be completely explained by batch effects. The SAS-AP dataset perform slightly better than the 1000 Genomes reference

panel consisting of BEB, GIH and PJL samples. So population substructure has a considerable effect on imputation accuracy.

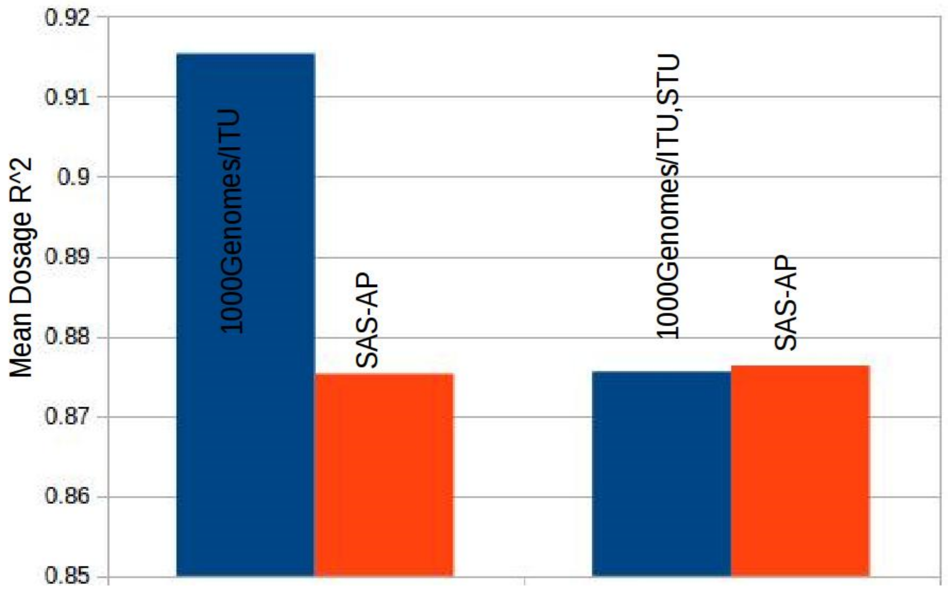

Figure S7.2: Mean Dosage R^2 of various imputation reference panels for a target set of 23 ITU samples in experiment 2.

S8. Commands for GATK pipeline for the SAS-AP data

Software Version:

GATK: 2.4-9
Samtools: 0.1.19
Picard: 1.97

****
Reference files:

Reference files were obtained from the GATK bundle b37 from GATK's website:
ftp://ftp.broadinstitute.org/
refPath="bundle/b37/human_g1k_v37.fasta"
dbSNP="bundle/b37/dbsnp_138.b37.vcf"
hapmap_vcf="bundle/b37/hapmap_3.3.b37.vcf"
omni_vcf="bundle/b37/1000G_omni2.5.b37.vcf"
mills_vcf="bundle/b37/Mills_and_1000G_gold_standard.indels.b37.vcf"
onekg_snp_vcf="bundle/b37/1000G_phase1.snps.high_confidence.b37.vcf"

****
Pipeline:

BAM File Analyses and Processing:

1. Build Bam index (picard: BuildBamIndex)

```
javaPath, picardPath, BuildBamIndex.jar, \
        'I=', bamPath+bam
```

2. Creating Realigner Target (gatk: RealignerTargetCreator)

```
javaPath, gatkPath, \
        '-T', 'RealignerTargetCreator', \
        '-I', bamPath+bam, \
        '-R', refPath, \
        '-o', workDirectory+bam[:-3]+'intervals', \
        '-nt', str(threads)
```

3.Realigning Indel (gatk: IndelRealigner)

```
javaPath, gatkPath, \
        '-T', 'IndelRealigner', \
        '-I', bamPath+bam, \
        '-R', refPath, \
        '-compress 0 ', \
        '-targetIntervals', workDirectory+bam[:-3]+'intervals', \
        '-o', workDirectory+bam[:-3]+'realigned.bam'
```

4. Marking Duplicates (samtools: rmdup)

```
javaPath, picardPath+'MarkDuplicates.jar',\
        'I='+workDirectory+bam[:-3]+'realigned.bam', \
        'O='+workDirectory+bam[:-3]+'realigned.marked.bam'
        'M='+workDirectory+bam+'.metrics'
```

5. Creating Bam Index (picard: BuildBamIndex)

```
javaPath, picardPath, BuildBamIndex.jar, \
        'I=', workDirectory+bam[:-3]+'realigned.marked.bam'
```

6. Base Recalibrating (gatk: BaseRecalibrator)

```
javaPath, gatkPath, \
        '-T', 'BaseRecalibrator', \
        '-I', workDirectory+bam[:-3]+'realigned.marked.bam', \
        '-o', workDirectory+bam+'.grp', \
        '-R', refPath, \
        '-knownSites', dbSNP, \
        '-nct', str(threads) \
```

7. Printing Recalibrated BAM (gatk: PrintReads)

```
javaPath, gatkPath, \
        '-T', 'PrintReads', \
        '-I', workDirectory+bam[:-3]+'realigned.marked.bam', \
        '-BQSR', workDirectory+bam+'.grp', \
        '-o', workDirectory+bam[:-3]+'recalibrated.bam', \
        '-R', refPath, \
        '-nct', str(threads) \
```

8. Variant calling (gatk: UnifiedGenotyper)

```
javaPath, gatkPath, \
        '-T', 'UnifiedGenotyper', \
        '-o', workDirectory+'output.vcf', \
        '-R', refPath, \
        '--dbsnp', dbSNP, \
        '-glm', 'BOTH' \
```

9. Variant Recalibration (gatk: VariantRecalibrator)

```
javaPath, gatkPath, \
        '-T', 'VariantRecalibrator', \
        '-R', refPath, \
        '-input', workDirectory+'output.vcf', \
        '-mode', 'SNP', \
        '-an QD -an HaplotypeScore -an MQRankSum -an ReadPosRankSum -an FS -
an MQ -an InbreedingCoeff', \
        '-recalFile', workDirectory+'output.SNP.recal', \
        '-tranchesFile', workDirectory+'output.SNP.tranches', \
```

```
        '-rscriptFile', workDirectory+'output.SNP.plots.R', \
        '-resource:hapmap,known=false,training=true,truth=true,prior=15.0', hapmap_vcf,
\
        '-resource:omni,known=false,training=true,truth=false,prior=12.0', omni_vcf, \
        '-resource:dbsnp,known=true,training=false,truth=false,prior=6.0', dbSNP

javaPath, gatkPath, \
        '-T', 'VariantRecalibrator', \
        '-R', refPath, \
        '-input', workDirectory+'output.vcf', \
        '-mode', 'INDEL', \
        '--maxGaussians 4', \
        '--minNumBadVariants 1000', \
        '-resource:mills,known=false,training=true,truth=true,prior=12.0', mills_vcf, \
        '-resource:dbsnp,known=true,training=false,truth=false,prior=2.0', dbSNP, \
        '-an DP -an FS -an ReadPosRankSum -an MQRankSum -an InbreedingCoeff', \
        '-recalFile', workDirectory+'output.INDEL.recal', \
        '-tranchesFile', workDirectory+'output.INDEL.tranches', \
        '-rscriptFile', workDirectory+'output.INDEL.plots.R' \

javaPath, gatkPath, \
        '-T', 'ApplyRecalibration', \
        '-R', refPath, \
        '-input', workDirectory+'output.vcf', \
        '--ts_filter_level 99.0', \
        '-tranchesFile', workDirectory+'output.SNP.tranches', \
        '-recalFile', workDirectory+'output.SNP.recal', \
        '-mode', 'SNP', \
        '-o', workDirectory+'output.recalibrated.SNP.vcf', \
        '-nt', str(threads)  \

javaPath, gatkPath, \
        '-T', 'ApplyRecalibration', \
        '-R', refPath, \
        '-input', workDirectory+'output.recalibrated.filtered.SNP.vcf', \
        '--ts_filter_level 99.0', \
        '-tranchesFile', workDirectory+'output.INDEL.tranches', \
        '-recalFile', workDirectory+'output.INDEL.recal', \
        '-mode', 'INDEL', \
        '-o', workDirectory+'output.recalibrated.SNP.INDEL.vcf', \
        '-nt', str(threads)
```

References

1. Lander ES, Waterman MS: **Genomic mapping by fingerprinting random clones: a mathematical analysis.** *Genomics* 1988, **2:**231-239.
2. The 1000 Genomes Project Consortium: **An integrated map of genetic variation from 1,092 human genomes.** *Nature* 2012, **491:**56-65.
3. Xing J, Watkins WS, Hu Y, Huff CD, Sabo A, Muzny DM, Bamshad MJ, Gibbs RA, Jorde LB, Yu F: **Genetic diversity in India and the inference of Eurasian population expansion.** *Genome Biol* 2010, **11:**R113.
4. Korneliussen TS, Albrechtsen A, Nielsen R: **ANGSD: Analysis of Next Generation Sequencing Data.** *BMC Bioinformatics* 2014, **15:**356.
5. Fumagalli M, Vieira FG, Linderoth T, Nielsen R: **ngsTools: methods for population genetics analyses from next-generation sequencing data.** *Bioinformatics* 2014, **30:**1486-1487.
6. Fumagalli M, Vieira FG, Korneliussen TS, Linderoth T, Huerta-Sanchez E, Albrechtsen A, Nielsen R: **Quantifying population genetic differentiation from next-generation sequencing data.** *Genetics* 2013, **195:**979-992.
7. Skotte L, Korneliussen TS, Albrechtsen A: **Estimating individual admixture proportions from next generation sequencing data.** *Genetics* 2013, **195:**693-702.
8. Nielsen R, Korneliussen T, Albrechtsen A, Li Y, Wang J: **SNP calling, genotype calling, and sample allele frequency estimation from New-Generation Sequencing data.** *PLoS One* 2012, **7:**e37558.
9. Browning BL, Browning SR: **Genotype imputation with millions of reference samples.** *The American Journal of Human Genetics* 2016, **98:**116-126.
